# Fine mapping and characterization of Fusarium wilt (*Fusarium oxysporum* f. sp. *benincasae*) resistance gene *Fob1(t)* in wax gourd (*Benincasa hispida* Cogn.)

**DOI:** 10.3389/fpls.2025.1555316

**Published:** 2025-05-26

**Authors:** Fahuo Li, Jian Qin, Jingying Li, Liang Cheng, Xuehan He, Xiaohui Zhong, Yangpeng Mo, Han Liang, Peng Wang, Yan Li, Yongguan Wu

**Affiliations:** ^1^ Vegetable Research Institute, Guangxi Academy of Agricultural Sciences, Nanning, China; ^2^ Guangxi Key Laboratory of Vegetable Breeding and New Technology Development, Nanning, China; ^3^ College of Agriculture, Guangxi University, Nanning, China

**Keywords:** wax gourd, Fusarium Wilt, bulked segregant analysis sequencing (BSA-seq), gene mapping, marker-assisted selection (MAS)

## Abstract

Fusarium wilt (FW), caused by the plant fungus *Fusarium oxysporum*, causes severe economic losses in wax gourd (*Benincasa hispida* Cogn.). Developing disease-resistant varieties is an effective measure. Currently, no reports exist on gene localization or cloning of genes associated with FW resistance in wax gourd. However, our team has identified an FW resistance gene and plans to apply it in resistant varieties. In this study, we used bulked segregant analysis sequencing and quantitative trait locus detection on 1,304 inbred lines derived from resistant GD68 and susceptible HM25 parents to identify FW resistance genes. We successfully identified a resistance locus between the 3M13.385 and 3M16.869 markers on chromosome 3, named *Fob1(t)*. Fine mapping between markers 3M15.904 and 3M16.373 (469 kb apart) identified 22 candidate genes. The transcriptome, sequencing comparison, and qRT-PCR analyses suggested that the endochitinase gene *Bch03G006380* is the resistance gene, with significant expression differences between the parents and a one-base mutation in the first exon. The study also revealed the roles of *Fob1(t)* in plant hormones, transcription factors, phenylpropane metabolism, oxidation–reduction, disease progression, enzymes, and cell wall modification pathways, enhancing FW resistance in GD68. Finally, we identified two closely linked insertion–deletion markers that can assist in the transfer and utilization of the *Fob1(t)* gene, significantly improving the screening rate of positive individual plants and reducing breeding time.

## 1 Introduction

Wax gourd (*Benincasa hispida* Cogn.) is an annual vine herb, rich in various kinds of supplements, including vitamins, nutrients, and seven essential amino acids for the human body (not including tryptophan) (Chu et al., 2006). It is an important vegetable for dietary vegetables, light vegetables, and southern vegetables to the north, with great economic value. Considering its great economic value, this important dietary vegetable that originated from China and East India is always transported to the north of China. At present, the cultivation area of wax gourd in China exceeds 350,000 hm^2^, concentrated in Guangdong, Guangxi, and Hainan, among other areas, with an average yield of over 112,500 kg/ha (Jiang et al., 2013; Xie et al., 2020). In recent years, wax gourd has gradually become the preferred vegetable species to support industrial revitalization, offering advantages of strong adaptability, easy cultivation, resistance to heat and humidity resistance, high yield, ease of storage, and transportation resistance (Zhou et al., 2014; Shi et al., 2022). Nevertheless, an increase in mass planting of wax gourd resulted in the development of Fusarium wilt (FW), which can occur during the whole growth period, flowering, fruit sitting, and fruit expansion. Notably, the incidence rate is as high as 60% or above due to strong adaptability to temperature, sensitivity to humidity, and challenges in control and eradication (Shen, 2012; Jing et al., 2019). Previous studies demonstrated that these dreadful diseases caused huge economic losses to the wax gourd industry, becoming the bottleneck to the sustainable and healthy development of wax gourd (Kang et al., 2012).

*Fusarium oxysporum*, a pathogenic fungus with devastating effects on plants, is known to induce FW in a variety of significant melon crops. The localization, cloning, and elucidation of the resistance mechanisms to FW in cucurbits have been progressing at a slow pace, with only 11 resistance genes having been documented thus far. In some instances, it was demonstrated that the main genes of the watermelon included *Fo-1.1*, *Fon-1*, and *qFon1-9* (Lambel et al., 2014; Zhang et al., 2013; Li et al., 2017; Branham et al., 2019, 2020). In addition, the major genes in cucumber include *Foc2.1*, *Foc4*, and *fw2.1* (Zhang et al., 2014; Zhou et al., 2015; Dong et al., 2019). Notably, FW resistance genes on the melon have been successfully identified, including *Fom-1*, *Fom-2*, *Fom-3*, and a newly located gene on chromosome 9 (Risser et al., 1976; Brotman et al., 2013; Joobeur et al., 2004; Zink and Gubler, 1985; Yang et al., 2024). However, no genes associated with FW resistance have been identified in wax gourd, and only five FW resistance varieties of wax gourd are currently in circulation within the market. Accordingly, the Sichuan Academy of Agricultural Sciences selected the pink wax gourd “ChuanFen No. 1” (Zeng et al., 2006) and the Guangdong Academy of Agricultural Sciences selected “TieZhu No. 1” and “TieZhu No. 2” (Xie et al., 2009a, 2013, 2017) for cultivation. To this end, “GuiShu No.6” and “GuiShu No.8” are being cultivated by the Guangxi Academy of Agricultural Sciences (Zhao et al., 2017; Wu et al., 2019; He et al., 2021). The predominant reasons include the medium- and low-resistance germplasm materials of wax gourd, as well as ancient local varieties with poor agronomic properties. Notably, these ancient germplasm materials cannot be directly bred and utilized (Liang et al., 2018; Zhou and Everts, 2001; Peng et al., 2020; Zeng et al., 2024). Moreover, the genetic basis of FW resistance in wax gourd is complex. For instance, Xie et al. (2009b) observed that FW resistance was controlled by multigene recessive inheritance, conforming to the “additive-dominant” model. Although the additive effect was predominant, susceptibility to the disease was partially dominant over resistance. Thus, it is of great significance to collect and screen more resistance sources and carry out the mining and utilization of the FW resistance gene of wax gourd.

Considering the slow research progress on wax gourd’s resistance to FW, this study aimed to explore gene mining and variety cultivation. According to the analysis of the parents (resistant GD68 and susceptible HM25), F_1_, and the genetic population (HM25/GD68 F_2_) by bulked segregant analysis sequencing (BSA-seq) and quantitative trait locus (QTL) detection, we successfully identified a resistance locus. Further fine mapping (screening recombinant individual plants and encrypted molecular markers), candidate gene analysis (including sequence difference, gene expression, and transcriptome analyses), and molecular markers were developed to assist in applying the resistance gene. Together, we believe that the FW resistance gene mapping and the development of molecular markers will certainly provide a research basis for exploring the mechanism of FW resistance and breeding for disease resistance.

## 2 Materials and methods

### 2.1 Plant materials and phenotypic evaluation

#### 2.1.1 Plant materials

In this study, wax gourd varieties, including GD68 with a high resistance to FW and HM25 with a high susceptibility to FW, were initially selected respectively as the male and female parents to construct an F_2_ segregating population. Further, the F_2:3_ line obtained from the F_2_ generation was used to screen recombinant individual plants for fine mapping analysis. In spring 2023, two parents (GD68 and HM25) and 1,304 individual plants (F_2_ population) were tested for FW. The wax gourd seeds were soaked in warm water for 6 h, wrapped in a towel, and then placed in a 28°C light incubator. After 2~3 days, the activated seeds were sowed in the seedling tray (0.5 m × 1.2 m) filled with substrate and grown to two true leaves for *F. oxysporum* treatment.

#### 2.1.2 Pathogen preparation

Initially, the DG510 strain (*F. oxysporum* Schl. f. sp. *benincasae*) was isolated, identified, and preserved by our team (Kang et al., 2012). Further, the culture medium was prepared using PS nutrient solution (200 g potatoes, 20 g sucrose, and 1,000 mL water), and cultured at 30°C for 6 days in the incubator. After producing a large number of spores, the concentration of the pathogen spores was calculated using a hemocytometer. The spore solution was prepared at a density of 5 × 10^5^ spores/mL.

#### 2.1.3 Phenotypic evaluation

The wax gourd seedlings were infected by the root irrigation method. First, the spore solution of the DG510 strain was irrigated to the roots of the wax gourd seedlings until the fungus solution overflowed from the cultivation plate. Then, all plants were transplanted in the plastic bucket (h = 0.2 m, d = 0.2 m) and planted at the experimental base in Nanning, Guangxi, PR China (108°22′E and 22°48′N). Further, the conventional production management was performed by timely watering and exposure to the net sun during drought, ensuring the normal growth of seedlings.

#### 2.1.4 Observed the disease occurrence of seedlings every day

When the highly susceptible material HM25 was dead, the disease characteristics were recorded, and a disease resistance score (DRS) was given for each seedling according to the FW classification of wax gourd (Zeng et al., 2004). Notably, the disease index (DI) of each material was calculated according to Equation 1 based on the survey and statistical results of the incidence of every individual plant,


(1) $$DI=\frac{\sum (s\times n)}{N\times S}\times 100$$

where s presents representative values for each disease grade, n indicates the number of diseased plants at each disease grade, N presents the total number of investigations, and S shows the representative value of the highest disease level.

### 2.2 Gene mapping

#### 2.2.1 DNA extraction

After 3 days of FW infection, the fresh young leaves from parents, F_1_, and F_2_ were initially collected in a 2-mL centrifuge tube and stored at −80°C. Further, DNA was extracted using the improved cetyl trimethyl ammonium bromide (CTAB) method (An et al., 2011). After extraction, the concentration of the DNA was determined using an ultra-micro spectrophotometer. Then, the quality of DNA was examined through 1.0% agarose gel electrophoresis.

#### 2.2.2 BSA-seq

Extreme resistance plants (n = 30) derived from the F_2_ population were selected, and the genomic DNA of each sample was diluted to 100 ng/μL. Then, 20 μL of DNA from each sample was mixed to develop one resistance DNA pool, which was also used to establish the susceptible DNA pool. Then, samples (two parents and two extreme DNA pools) were sent to Shanghai PersonalBio Technology Co., Ltd. (Shanghai, China) for BSA-seq to search for resistance loci and the candidate intervals (Michelmore and Kesseli, 1991). Further, the association analysis was conducted between a resistance-susceptible mixed pool and two-parent pools with GX-19 (unpublished) as the reference genome. Single-nucleotide polymorphism (SNP) and insertion–deletion (InDel) were detected using the GATK software. The candidate intervals were analyzed using the ED, GPS, SNP-index, and MutMap algorithm.

#### 2.2.3 QTL validation

Considering the results of the four analytical models, InDel molecular markers covering the target region on the corresponding chromosome were designed according to the BSA-seq data. The polymorphic markers were detected in the two extreme DNA pools and two parents. Furthermore, several polymorphic markers were identified between the parents in the target chromosomal region. In the F_2_ populations, the bands consistent with the resistant parents (GD68) or susceptible parents (HM25) were designated as B and A, respectively. The samples with both bands of resistant and susceptible parents were designated as H. The markers with polymorphism on both parents were tested for genotype on 96 samples (select representative plants from 1,304 F_2_ populations). Then, these genotypes were combined with phenotype to detect resistance locus with composite interval mapping using the QTL IciMapping software (Wang, 2013). Finally, the BSA-seq resistance locus was validated when the threshold limit of detection (LOD) value ≥2.5.

#### 2.2.4 Fine mapping

Based on the interval position of QTL mapping, markers on both sides of the localization were selected to screen individual recombinant plants in the F_2:3_ lines, as well as to identify the DI phenotype by Equation 1 for the recombinant individuals, develop new primers, and obtain the genotypes of these recombinant individuals using the newly designed primers. The primer development was aimed at specific QTL localization intervals using BLAST tools from websites such as the National Center for Biotechnology Information (NCBI). By comparing the genomic sequences of GX-19 as the reference genome, primers with product sizes between 100 and 300– bp were designed using the Premier Primer software (version 5.0) for insertion and deletion regions of over 10 bp. The primer BLAST tool in the NCBI was used to compare the designed primers, and primers with high specificity were selected as candidates and synthesized by Huada Gene Technology Co., Ltd. (Shenzhen, China).

### 2.3 RT-qPCR and transcriptome analyses

The plant samples (GD68 and HM25) infected with FW were taken at five different time points (0, 4, 6, 8, and 10 days). The first true leaf of the seedlings was rapidly collected and stored in liquid nitrogen or −80°C freezer for RT-qPCR and transcriptome analyses. It should be noted that each treatment time point was performed in triplicate.

#### 2.3.1 RT-qPCR analysis

Initially, RNA was extracted using TaKaRa TransZol Up Plus RNA Kit (Tokyo, Japan) and then reverse transcribed to complementary DNA (cDNA) using the PrimeScript™ First-Strand cDNA Synthesis SuperMix kit. Further, the RT-qPCR primers were designed based on the GX-19 genome using the Primer 5.0 software for candidate genes within the mapping interval. The PCR analysis was performed on the ABI PRISM 7300 real-time PCR system (Applied Biosystems, Waltham, MA, USA). According to the instructions of the PrimeScript™ RT kit (TaKaRa Co., Ltd.), the RT-qPCR system was prepared using 0.4 µL of forward and reverse primers, 0.4 µL of Passive Reference Dye (50×), 1 µL of template cDNA, 10 µL of 2× TransStart Top, and 20 µL of ddH_2_O. The reaction procedure involved preheating for 30 seconds at 94°C and for 40 cycles of 5 seconds at 94°C, 15 seconds at 56°C, and 10 seconds at 72°C. The samples were set up in triplicate. Further, the standard curve was drawn to calculate the slope and R^2^. The wax gourd gene Actin (upstream primer 5′-CCATTCCCACCTCGTGTTCA-3′ and downstream primer 5′-TCCCACAGTTTCCTCACAGC-3′) was used as a reference to analyze the gene expression level between samples by the 2^−ΔΔCt^ method. Based on the quantitative expression results of three replicates, the changes in the gene expression of the target candidate gene were compared at various time points of FW treatment (Cheng et al., 2021).

#### 2.3.2 Transcriptome sequencing

Initially, the samples collected at different time intervals were sent to Shanghai Majorbio Bio-Pharm Technology Co., Ltd. (Shanghai, China) for eukaryotic mRNA sequencing. The experimental procedures sequentially included extracting total RNA, enriching mRNA, mRNA fragmentation, reverse synthesis of cDNA, adapter ligation, construction of sequencing library, and high-throughput sequencing. The NovaSeq X Plus and DNBSEQ-T7 platforms were used for sequencing, and dual-end reads were obtained. The Fastp software was employed to perform data quality filtering and obtain clean reads. Using the TopHat2 software (Kim et al., 2013), paired clean reads were aligned with the wax gourd reference genome GX-19 to obtain mapped data. The Cufflinks or StringTie software was used to assemble and splice mapped reads (Trapnell et al., 2010; Pertea et al., 2015). Based on the length of the gene and the Htseq count, the fragments per kilobase of transcript sequence per million base pair sequence (FPKM) and transcripts per million reads (TPM) were calculated to quantify the gene expression for gene/transcript length and sequencing depth. To compare genomes (Li and Dewey, 2011), read counts for each sample gene/transcript were obtained using RNA-seq by Expectation–Maximization (RSEM). Finally, FPKM or TPM conversion was performed to obtain standardized gene/transcript expression levels.

#### 2.3.3 Differential expression, enrichment, and functional annotation of differentially expressed genes

Difference multiple | log2 (Fold Change) | > 2 and p < 0.01 were used as criteria to screen differentially expressed genes (DEGs). Differential analysis was performed using the DESeq2 software of DEGs with non-redundant protein sequences (NR) database (https://www.ncbi.nlm.nih.gov/) (Love et al., 2014), SwissProt protein sequences (SWISS-PROT) database (https://www.uniprot.org/uniprotkb/), eukaryotic homologous protein cluster (KOG) (https://www.ncbi.nlm.nih.gov/COG/), Pfam database (https://www.ebi.ac.uk/interpro/), the Gene Ontology (GO) (https://geneontology.org/), and Kyoto Encyclopedia of Genes and Genomes (KEGG) databases (https://www.kegg.jp/) for comparison. The information on the functional annotation and pathway enrichment of the DEGs was obtained.

### 2.4 Molecular marker-assisted breeding

Two InDel markers tightly linked to the predicted main regulatory resistant gene were designed using Premier 5.0 (**Supplementary Table S1**). Combined with developed markers, 13 wax gourd germplasms from the parent plants (GD68 and HM25), nine F_2_ individual plants, and four negative wax gourd materials (149, 158, 183, and 121-long) without the resistant gene were used for molecular marker-assisted and FW resistance accuracy verification experiments.

### 2.5 Statistical analysis

The expressed data were compared using one-way analysis of variance (ANOVA) to compare multiple samples and the t-test to examine the differences between the two groups. All the data were compared using the least significant difference (LSD) test at the 5% or 1% significance level.

## 3 Results

### 3.1 Genetic analysis of GD68 resistance

**Figure 1:**
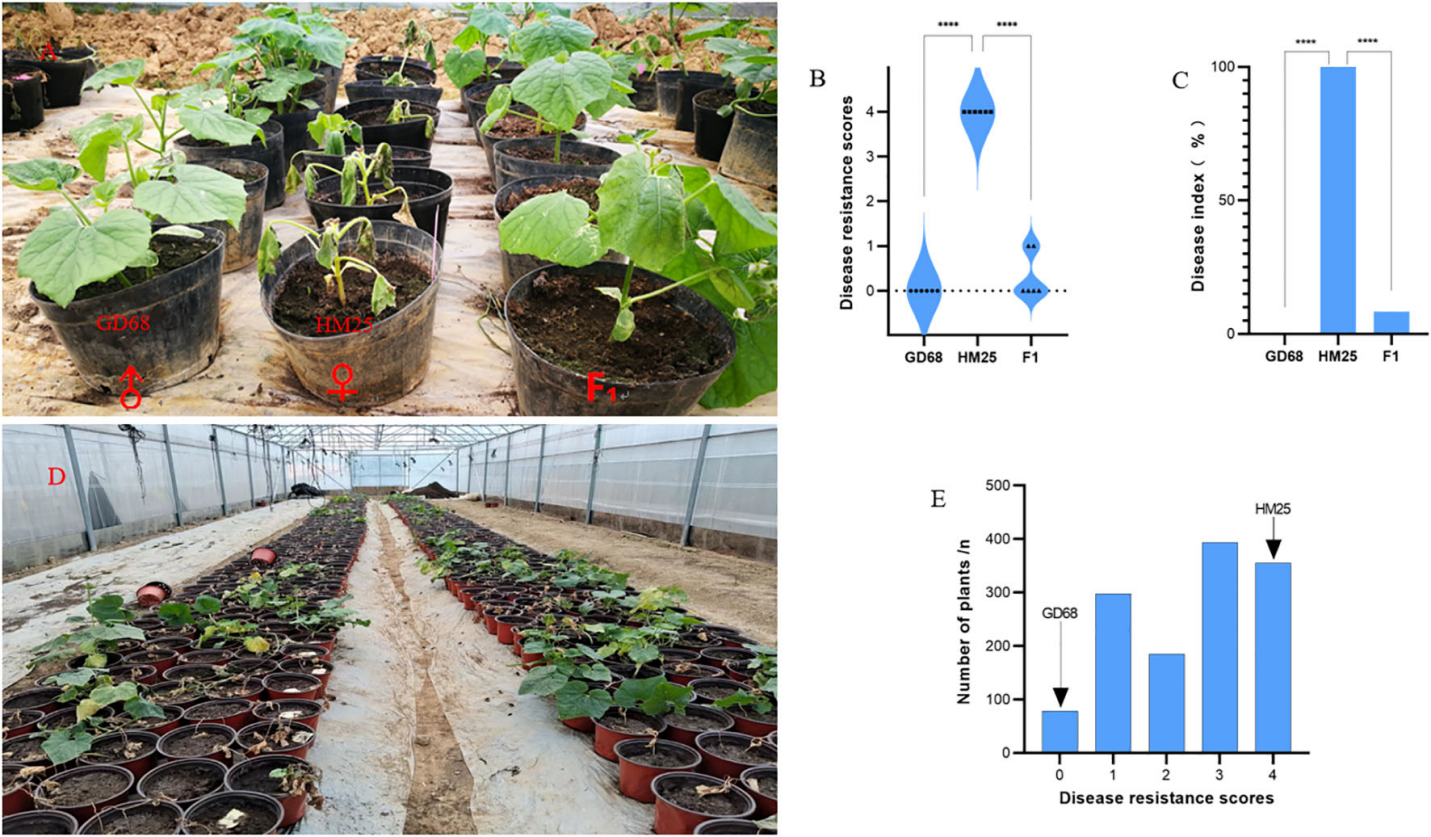
GD68 resistance identification and genetic characteristics. **(A)** The bright field image shows the phenotype of Fusarium wilt (FW) identification at the seedling stage and corresponding **(B)** disease resistance score and **(C)** disease index, where from the left only GD68, HM25, and HM25/GD68 F_1_ populations had six replicates for each material. The graphs show **(D)** the HM25/GD68 F_2_ population phenotype of FW identification at the seedling stage, with a total of 1,304 individual plants and corresponding **(E)** disease resistance score. The left arrow indicates the resistance value of GD68, and the right arrow indicates the resistance value of HM25.

After 14 days of infection with DG510 pathogen (*F. oxysporum* f. sp. *benincasae*), the leaves of susceptible parent HM25 plants showed wilting, brown and decaying stems, lodging of the whole plant, and death, with a DRS of 5 and DI of 100. Contrarily, the leaves of resistant parent GD68 plants were healthy, showing normal stems and vigorous vitality, with DRS = 0 and DI = 0. Notably, it showed a significant difference from HM25. In addition, the same experiment was conducted on F_1_ plants, indicating healthy leaves with only individual leaves wilted or yellow stemness health (DRS = 0 and DI = 8.3). The overall states showed not much difference from GD68, which, however, showed a significant difference with HM25 (**Figures 1A–C**). It was observed that HM25 was a highly susceptible material. At the same time, GD68 was a highly resistant material, and F_1_ was significantly resistant to FW, indicating that the resistance gene could be inherited dominantly. A total of 1,304 plants from the F_2_ population were identified for their resistance to FW. Notably, the distribution of their resistance grades basically followed a normal distribution. The ratio of the coefficient of resistant plants (DRS ≤ 3) and susceptible plants (DRS ≥ 4) was 955:349 (χ^2^ = 2.16 < χ^20.01,1^ = 3.84). The chi-square test validated the conformation to the segregation pattern of a major gene (**Figures 1D, E**).

### 3.2 Resistance locus detection and validation

**Figure 2:**
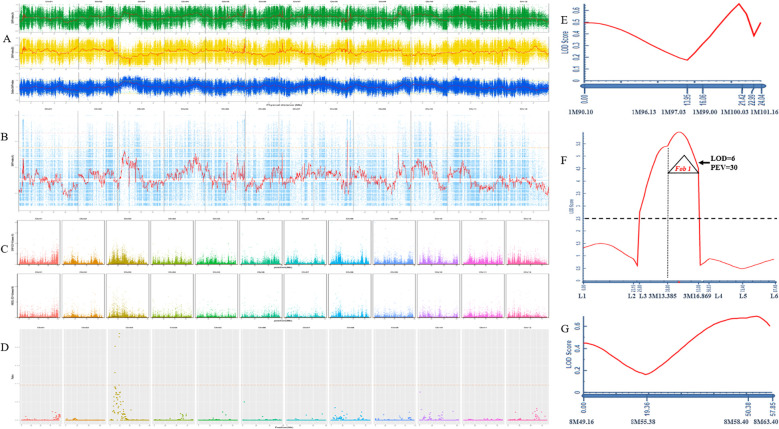
GD68 resistance locus detection and identification. **(A)** Single-nucleotide polymorphism (SNP) index model. The horizontal axis represents the names and lengths of each chromosome, the vertical axis represents the SNP index value, the red line represents the mean SNP index, the orange line represents the 99% confidence line, and the green line represents the 95% confidence line. The positioning range is Chr03:9.8Mb-20.4Mb. **(B)** MutMap model. The horizontal axis represents the names and lengths of each chromosome, the vertical axis represents the SNP index value, the red line represents the mean SNP index, the pink line represents the threshold line corresponding to the 99th percentile, and the orange line represents the threshold line corresponding to the 95th percentile. The positioning range is Chr08:56.9Mb-58Mb. **(C)** ED model. The horizontal axis represents the base position on each chromosome, and the vertical axis represents the Nth power of the ED value of SNP/insertion–deletion (InDel). Each point represents an SNP/InDel, with black indicating the fitting line and orange indicating the threshold line. The positioning range is Chr01:101.3Mb-96.2Mb Chr03:5.5Mb-33.4Mb. **(D)** GPS model. The horizontal axis represents the SNP position on the chromosome, and the vertical axis represents the corresponding ratio value [using the sliding window method for denoising, that is, calculating the ratio of the number of sites with −ln (p-value) values greater than 10 within the sliding window (400 kb) to the total number of sites within the sliding window; the larger the value, the more associated it is with the trait]. The positioning range is Chr03:10Mb-19.6Mb. The quantitative trait locus (QTL) detection on chromosomes 3 **(E)**, 1 **(F)**, and 8 **(G)**. The X-axis is genetic distance (cM) and corresponding molecular markers. The Y-axis is the limit of detection (LOD) value; dashed line LOD = 2.5; LOD ≥ 2.5 indicates the presence of a major effector locus. PEV explains the phenotypic variation caused by this locus.

Further, 30 extremely resistant and 30 susceptible plants from HM25/GD68 F_2_ were selected to construct extreme resistance and susceptibility pools for BSA-seq analysis. With the analysis using the four models of detection, regions exceeding the 95% threshold line were selected as candidate regions for trait correlation. The ED model results showed that the candidate intervals were between Chr01:101.3Mb-96.2Mb and Chr03:5.5Mb-33.4Mb. The SNP index modeling displayed that the candidate interval was Chr03:9.8Mb-20.4Mb. The GPS model results showed that the candidate interval was Chr03:10Mb-19.6Mb, while the MutMap model showed that the candidate interval was Chr08:56.9Mb-58Mb (**Figures 2A–D**).

Considering the large range of detected loci, InDel markers on chromosomes 1, 3, and 8 were designed based on biparental resequencing data to cover the corresponding candidate interval. The genotypes of 1,304 HM25/GD68 F_2_ plants and the DRS of FW resistance phenotype were combined to construct a genetic map and detect resistance locus QTL. A significant QTL was detected between markers 3M13.385 and 3M16.869 on Chr03, LOD = 6, which could explain 30% of the genetic variation (**Figure 2E**). Based on the absence of a gene or loci identified for FW resistance in wax gourd, this locus was named *Fob1(t)*, following international naming rules. Meanwhile, no resistance sites on Chr01 and Chr08 were detected (**Figures 2F, G**).

### 3.3 Fine mapping of *Fob1(t)*

**Figure 3:**
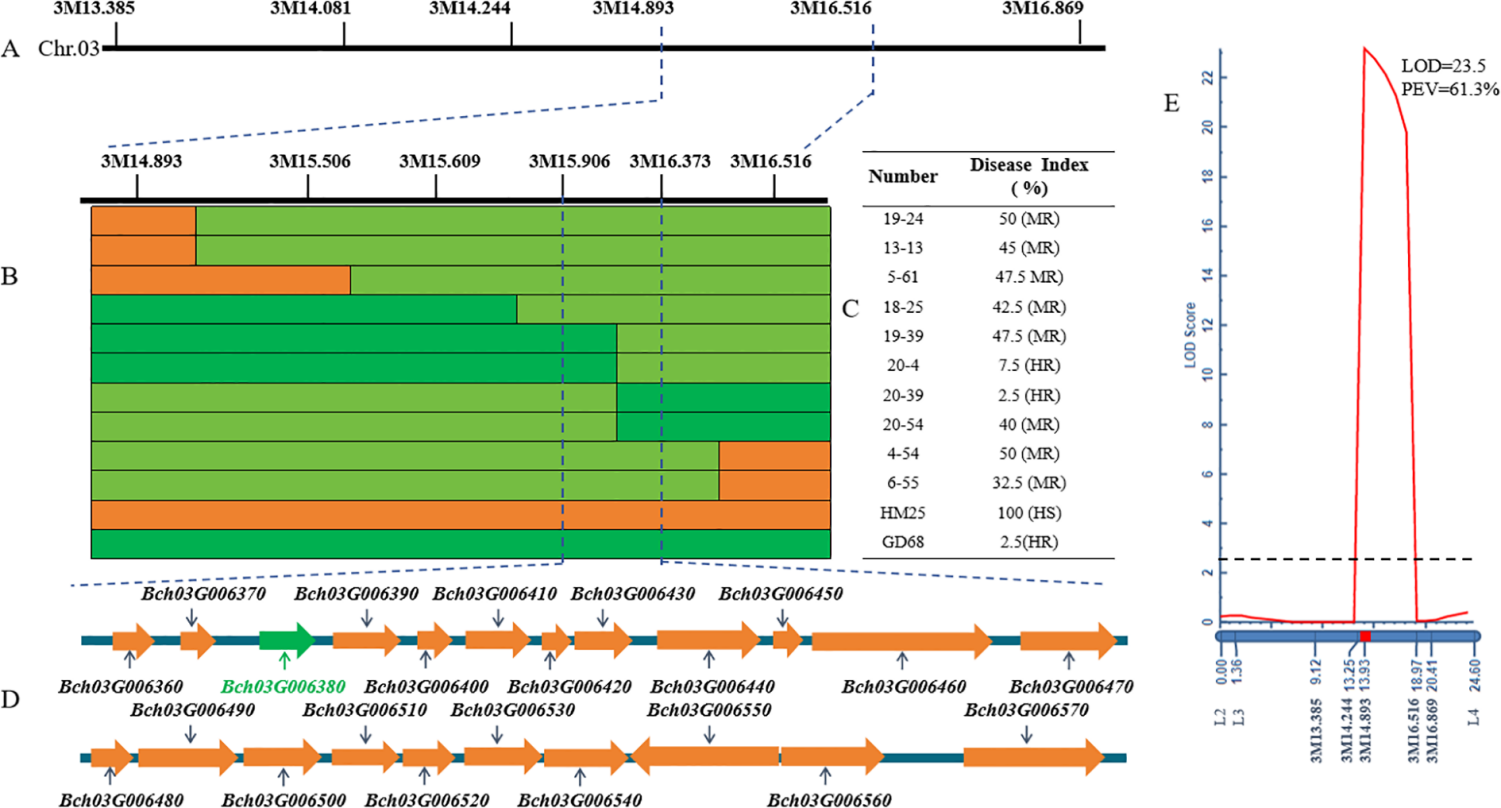
Fine mapping of *Fob1(t)*. **(A, B)** The images show that the markers correspond to specific locations on chromosome 03, as well as **(C)** genotypes and phenotypes of the selected recombinants. Green, yellow, and light green bars denote the resistant parent homozygote of GD68, susceptible parent homozygote of HM25, and heterozygotes, respectively. Other numbers refer to recombinants derived from the F_2:3_ line generations of HM25/GD68, and the DI phenotype of each recombination is calculated based on the Equation 1 of 10 individual plants of its offspring. **(D)** The image presents candidate genes of fine mapping interval, where green represents the most likely target gene. **(E)** Quantitative trait locus (QTL) mapping map of encryption mark on chromosome 3. The X-axis vertical is genetic distance (cM), and the horizontal is corresponding molecular markers. The Y-axis is the limit of detection (LOD) value; dashed line LOD = 2.5; LOD ≥ 2.5 indicates the presence of a major effector locus. PEV explains the phenotypic variation caused by this locus.

To finely map the resistance genes, several recombinants (n = 287) derived from 1,304 F_2_ plants were identified using markers 3M13.385 and 3M16.896. A total of six pairs of InDel markers (**Supplementary Table S1**) in the mapped interval were initially designed at 3.48-Mb intervals based on the QTL results. Further, a genotype–phenotype joint analysis was performed on the parents and recombinants. The interval was mapped to a region of 1.62 Mb between 3M14.893 and 3M16.516 (**Figures 3A, E**). To identify and localize the genes controlling resistance to FW, the F_2:3_ line of 3,000 plants was screened to retrieve additional recombinants with 3M14.893 and 3M16.516, finally obtaining 42 recombinants. Further, the development of four pairs of InDel markers between 3M14.893 and 3M16.516 resulted in the detection of the genotypes of the 42 recombinant individual plants. Moreover, two recombinants (19–39 and 20-4) could determine the right marker (3M16.373), and the other two recombinants (20–39 and 20-54) could choose the left marker (3M15.906) in the target region (**Figures 3B, C**). It was finally mapped to be one region harbored by markers (3M15.906 and 3M16.373), which was approximately 467 kb based on the wax gourd reference genome (GX-19). Accordingly, 22 genes were predicted in the target region (**Table 1**, **Figure 3D**).

**Table 1:** Predicted candidate genes for *Fob1* in wax gourd reference genome GX-19.

Gene ID	Annotation
*Bch03G006360*	Chitinase 1 [*Cucumis sativus*]
*Bch03G006370*	Endochitinase-like [*Cucurbita moschata*]
*Bch03G006380*	Endochitinase [*C. sativus*]
*Bch03G006390*	Pentatricopeptide repeat-containing protein *At2g17670* [*C. sativus*]
*Bch03G006400*	Uncharacterized protein *LOC101217557* [*C. sativus*]
*Bch03G006410*	PREDICTED: uncharacterized protein *LOC103485177* [*Cucumis melo*]
*Bch03G006420*	PREDICTED: uncharacterized protein *LOC103485178* [*C. melo*]
*Bch03G006430*	IST1-like protein isoform X2 [*C. sativus*]
*Bch03G006440*	Protein *S*-acyltransferase 10 isoform X1 [*C. sativus*]
*Bch03G006450*	Mediator of RNA polymerase II transcription subunit 12-like isoform X1 [*C. melo* var. *makuwa*]
*Bch03G006460*	Putative F-box protein *At1g65770* [*C. sativus*]
*Bch03G006470*	PREDICTED: putative F-box protein *At1g65770* [*C. melo*]
*Bch03G006480*	F-box protein SKIP23-like isoform X1 [*C. sativus*]
*Bch03G006490*	PREDICTED: UPF0548 protein *At2g17695* [*C. melo*]
*Bch03G006500*	PREDICTED: thiol-disulfide oxidoreductase LTO1 [*C. melo*]
*Bch03G006510*	Uncharacterized protein *LOC101216776* isoform X1 [*C. sativus*]
*Bch03G006520*	Ammonium transporter 3 member 3 [*C. sativus*]
*Bch03G006530*	Ankyrin repeat domain-containing protein, chloroplastic [*C. moschata*]
*Bch03G006540*	PREDICTED: serine/arginine-rich splicing factor SR45a [*C. melo*]
*Bch03G006550*	Phospholipase D delta [*C. melo* var. *makuwa*]
*Bch03G006560*	PREDICTED: probable prolyl 4-hydroxylase 10 [*C. melo*]
*Bch03G006570*	PREDICTED: aconitate hydratase [*C. melo*]

### 3.4 Candidate gene analysis

**Figure 4:**
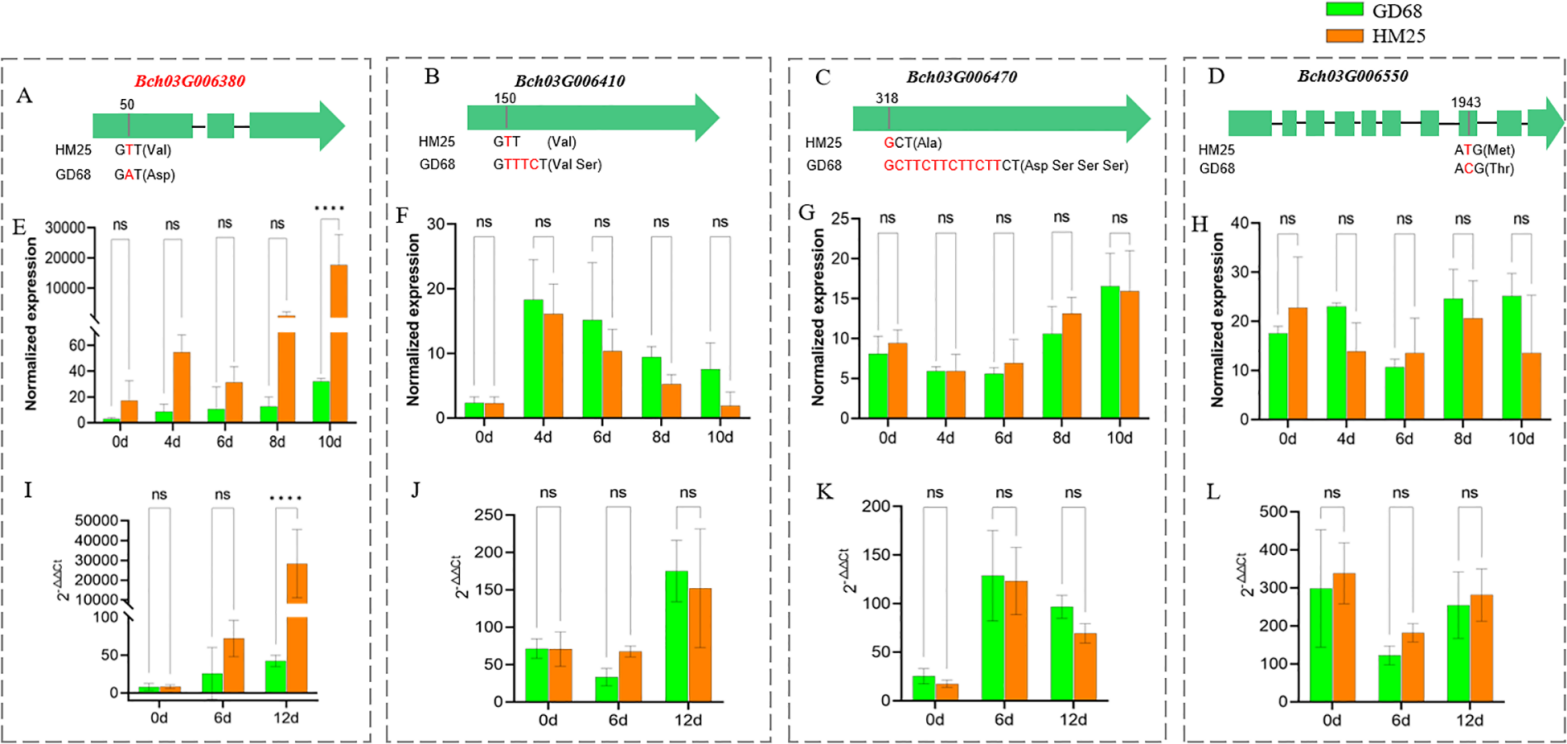
Candidate genes analysis of *Fob1(t)*. **(A–D)** The image shows the CDS comparison of *Bch03G006380*, *Bch03G006410*, *Bch03G006470*, and *Bch03G006550* between GD68 and HM25. Red represents differential bases, while parentheses indicate corresponding changes in amino acids. **(E**–**H)** The graphs show transcriptome validation of the four candidate genes in GD68 and HM25. Difference multiplelog2 (Fold Change)> 2 and p < 0.01 were used as criteria to screen differentially expressed genes (DEGs). Differential analysis was performed using the DESeq2 software. Bars represent the means of three replicates. Error bars represent the SD. ****, p < 0.0001. **(I**–**L)** The image shows quantitative reverse transcription polymerase chain reaction validation of the four candidate genes between GD68 and HM25. Actin was used as an internal control. The relative expression level of each gene was measured using the 2^−ΔΔCt^ method. Bars represent the means of three replicates. Error bars represent the SD. ****, p < 0.0001. ns, no significant difference.

To analyze the candidate genes, sequencing comparison, transcriptome, and RT-qPCR analyses were performed to validate the 22 genes from GD68 and HM25 (**Table 1**). It was observed that there were only four genes with sequence differences in the coding sequences (CDS) of two parents, GD68 and HM25. Consequently, *Bch03G006380* showed one SNP variation in the first exon (T changed to A), causing the amino acid Val to change to Asp (**Figure 4A**). *Bch03G006410* presented one variation of three base insertions in 5′UTR, causing no frameshift (**Figure 4B**). *Bch03G006470* showed one variation of 12 base insertions in 5′UTR, causing no frameshift (**Figure 4C**). *Bch03G006550* presented one SNP variation in the eighth exon (A changed to G), causing the amino acid Met to change to Thr (**Figure 4D**). After FW treatment, only gene *Bch03G006380* showed a significant difference in the expression level of transcriptome and RT-qPCR on the 10th day. Specifically, the expression level of the *Bch03G006380* gene from HM25 reached 15,000 (transcriptome) and 20,000 (RT-qPCR), which was significantly different from GD68 (**Figures 4E, I**). These findings indicated that it may belong to constitutive genes, and FW inoculation could not lead to excessive expression in resistant materials. While another 3 genes (*Bch03G006410*, *Bch03G006470*, and *Bch03G006550*) didn't showed a significant difference (**Figures 4F–H, J–L**). Together, these findings suggested that *Bch03G006380* was likely the target resistance gene, which could mediate an endochitinase gene and offer unique efficacy in antifungal treatment.

**Figure 5:**
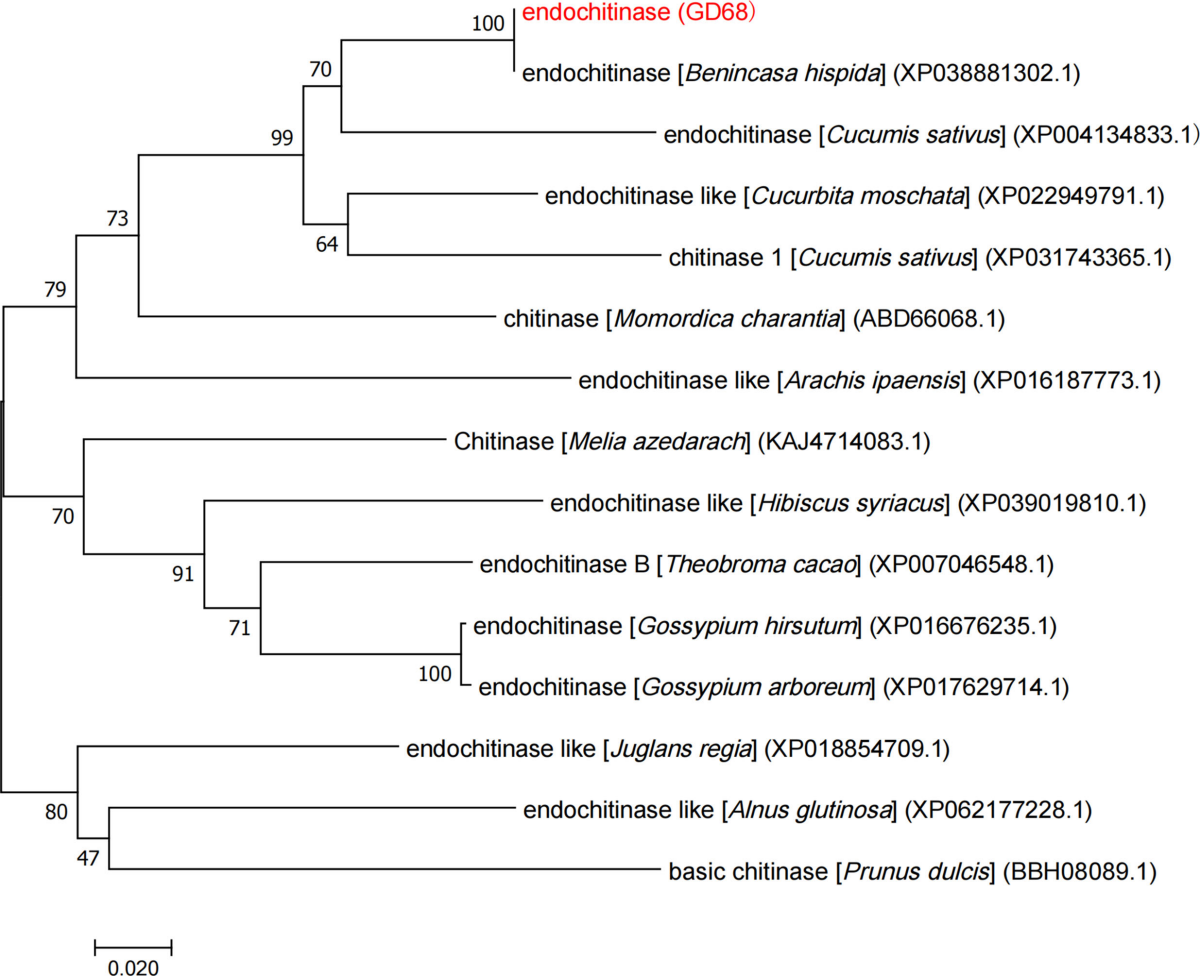
Phylogenetic tree for *Bch03G006380* and its homologous proteins. Phylogenetic tree was constructed with 1,000 bootstrap replicates using MEGA 7.0. Numbers at tree forks indicate bootstrap values.

To understand the relationship between the protein sequence of *Bch03G006380* and other homologous sequences, we analyzed the *Bch03G006380* protein sequence using NCBI BLAST (NCBI, Bethesda, MD, USA). Subsequently, we used the neighbor-joining function in MEGA 7.0 to generate a 1,000-repeat phylogenetic tree, using the bootstrap method. The results revealed that *Bch03G006380* shared a close phylogenetic relationship with cucurbits, including cucumbers, pumpkins, and bitter gourd (**Figure 5**).

### 3.5 Molecular pathway analysis of *Fob1(t)*

**Figure 6:**
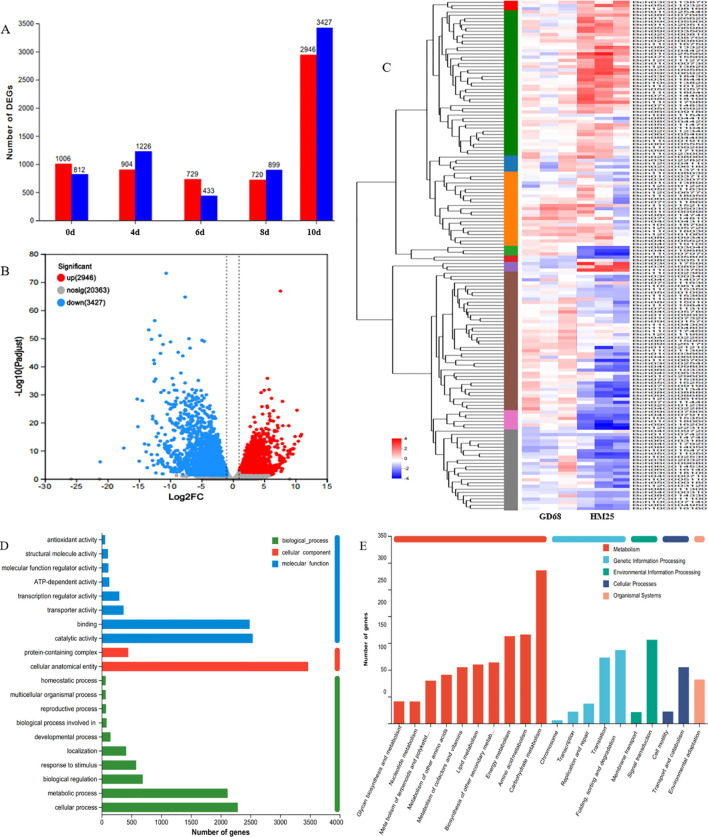
Molecular pathway analysis of *Fob1(t)*. **(A**–**E)** The images show the differential gene statistics diagram, volcano diagram, heatmap diagram, Gene Ontology (GO) annotation diagram, and Kyoto Encyclopedia of Genes and Genomes (KEGG) annotation diagram, with three types of biology per sample.

To explore the pathway mediated by the resistance gene *Fob1(t)*, RNA-seq was performed at five different time points (0, 4, 6, 8, and 10 days) after FW inoculation of the parent materials (GD68 and HM25). At 4, 6, and 8 days, the gene transcription levels between resistant and susceptible materials remained at a relatively stable level (**Figure 6A**). Contrarily, the gene transcription levels at 10 days between the resistant materials increased sharply (up = 2,946, down = 3,427) (**Figures 6A, B**). These results were consistent with the FW phenotype of wax gourd seedlings after treatment, indicating that obvious FW symptoms appeared in susceptible materials on the 10th day. Accordingly, the gene transcription levels between resistant and susceptible materials were significantly induced only when the wilt phenotype appeared after FW was treated. At 10 days, the differential genes were annotated. On the one hand, the GO analysis presented that these genes may mainly affect various functions, such as binding, catalytic activity, cellular anatomical entity, metabolic process, and cellular process (**Figure 6D**). On the other hand, the KEGG analysis demonstrated that these genes may affect other functions, such as energy metabolism, amino acid metabolism, carbohydrate metabolism, translation, folding, sorting, and degradation, as well as signal transduction (**Figure 6E**). These enrichment analyses related to disease resistance were mainly classified into seven categories: 307 genes related to plant hormones (*ABA*, *SA*, *MeJA*, *ET*, *IAA*, etc.), 289 genes related to transcription factors (*AP2/ERF*, *WRKY*, *MYB*, *bHLH*, *CRF*, *EFR*, etc.), 173 genes related to plant phenylpropane metabolism pathways (*PER*, *BGLU*, *PAL*, *4-CL*, *CCoAOMT*, etc.), 100 genes related to plant redox (*RBOH*s, *APX*, *CAT*, *POD*, *GPX*, etc.), 66 genes related to disease progression (*MLP*, *MLO*, and *TMVR*), 47 genes related to enzymes (*MAPK kinase*, *LRR-RLK*, *chitinase*, *cellulase*, etc.), and nine genes related to cell wall decoration (**Figure 6C**).

### 3.6 Development and application of molecular markers

**Figure 7:**
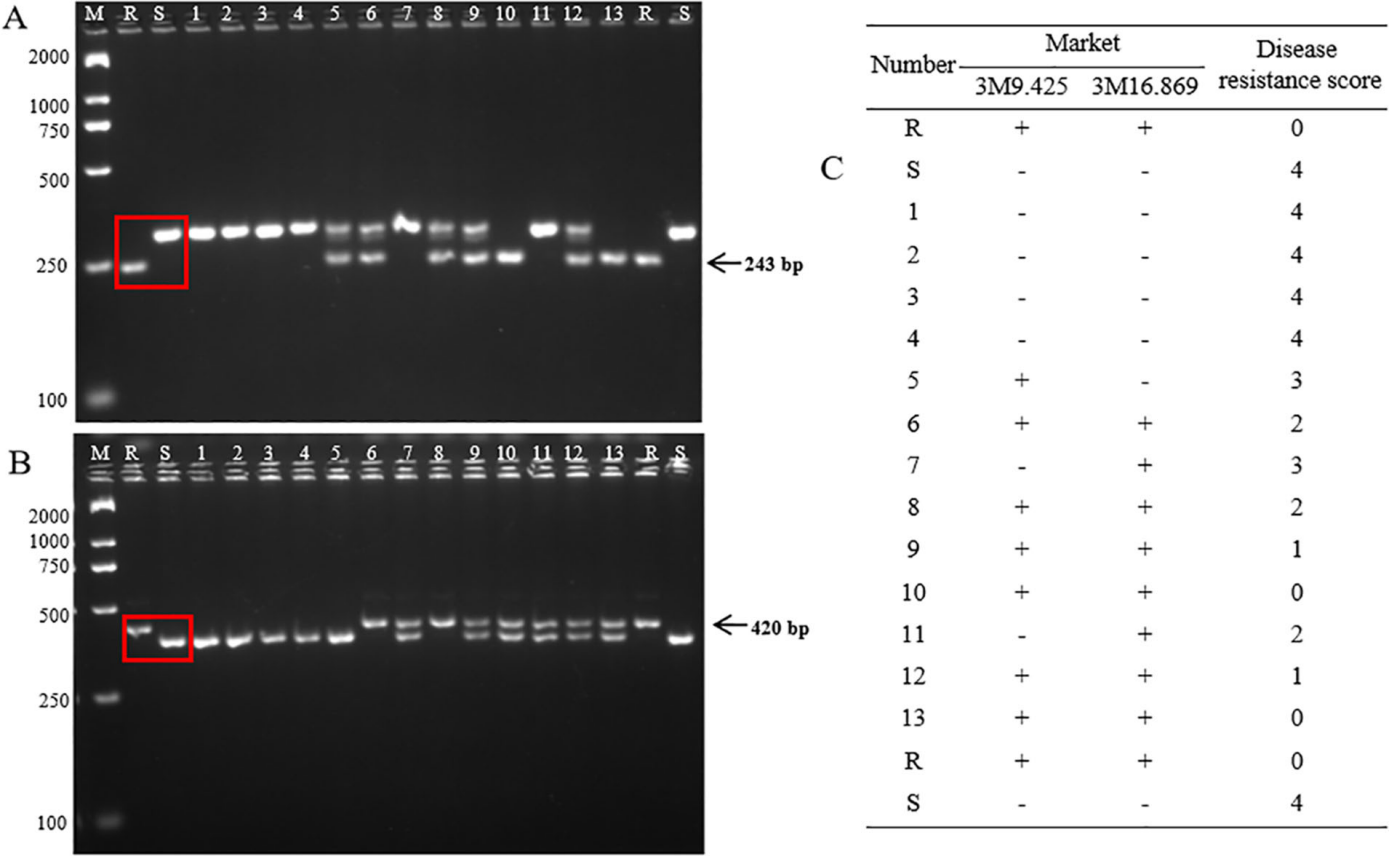
Marker-assisted selection detection. The images show the detection of markers 3M9.425 **(A)** and 3M16.869 **(B)** in 2.5% agarose gel. M refers to the BM2000 DNA marker, and the sizes of the bands from top to bottom are 2,000, 1,000, 750, 500, 250, and 100 bp, respectively. R indicates GD68, and S refers to HM25. Lanes 1–4 are four negative wax gourd materials without the *Fob1(t)* gene. Lanes 5–13 are partial HM25/GD68 F_2_ selected individual plants. The arrow indicates the target band of the resistant parent. **(C)** The table presents the resistance grading values of Fusarium wilt (FW) corresponding to “+” and “−”, where “+” indicates that the molecular test result is positive with the *Fob1(t)* gene and “−” the molecular test result is negative without the *Fob1(t)* gene.

Finally, two markers (3M9.425 and 3M16.869) were developed for the resistance genes *Fob1(t)* to apply marker-assisted selection (MAS). The InDel marker (3M9.425) showed a deletion of 58 bp in GD68 compared to HM25. After the PCR product was electrophoretically analyzed on an agarose gel, yielding one band of 243 bp in GD68, the susceptible varieties presented one band of approximately 301 bp (**Figure 7A**, lanes 1 and 2; **Supplementary Figure S1**). The InDel marker (3M16.869) possessed an insertion of 42 bp in GD68 compared to HM25. Although it yielded one band of approximately 420 bp in GD68, the susceptible varieties showed one band of approximately 378 bp (**Figure 7B**, lanes 1 and 2; **Supplementary Figure S2**).

These two markers were validated with 13 wax gourd accessions, including four susceptible varieties and nine HM25/GD68 F_2_ individuals’ plants. The bands of four susceptible varieties were consistent with HM25, whereas those of nine HM25/GD68 F_2_ individual plants were consistent with GD68. The result of the detected markers was consistent with FW resistance identification (**Figure 7C**). Together, these findings indicated that two markers could be used for MAS.

## 4 Discussion

*F. oxysporum*, which is the main cause of melon wilt, is a worldwide soil pathogen fungus with a wide host range. Currently, two resistance genes have been cloned on melon, *Fom-1* and *Fom-1*, both of which belong to R-type protein, where *Fom-1* is an NB-ARC protein and *Fom-2* is an NBS-LRR protein (Brotman et al., 2013; Joobeur et al., 2004). In addition, some FW resistance genes are also mapped in cucurbits. QTL *fw2.1* was accurately located within the 0.60-Mb interval and transcriptome screening analysis revealed five genes were significantly induced by FW (calmodulin protein, an edited transmembrane protein, and a serine-rich protein, among others) (Dong et al., 2019). In another instance, Wang and colleagues conducted a genome-wide association analysis of 192 bitter gourd germplasms for FW and analyzed candidate genes; potassium transporter 5-like (encoded by *LOC111013191*) was homologous to Arabidopsis CCC1 protein (proven to enhance Arabidopsis resistance to *Pseudomonas syringae*), which may be a candidate gene for resistance to FW (Wang, 2022). In this study, a major gene was identified for resistance to FW in wax gourd, currently inferred to be an edited endogenous chitinase gene *Bch03G006380*. The expression level of *Bch03G006380* showed significant differences after being infected by FW, indicating that it could mediate the synthesis of chitin-related genes and substances. The transcriptome analysis revealed that *Fob1(t)* could participate mainly in seven ways related to disease resistance, namely plant hormones, transcription factors, phenylpropane metabolism, oxidation–reduction, disease progression, enzymes, and cell wall modification, enhancing FW resistance in GD68. Nevertheless, the specific regulation mechanism remains to be further validated.

MAS is an effective molecular breeding technique widely used in breeding programs for FW resistance. Currently, the common markers include Cleaved Amplified Polymorphic Sequences/derived Cleaved Amplified Polymorphic Sequences (CAPS/dCAPS), Simple Sequence Repeat (SSR), and InDel marker. These markers can assist in the screening of positive individual plants in breeding and greatly accelerate the process of breeding. For instance, Zhang and colleagues developed three linked CAPS/dCAPS markers for the watermelon variety ‘Calhourn Gray’ FW resistance gene *Fon-1*. In contrast, Li and colleagues developed an InDel marker closely linked to the disease-resistance gene *Fon-1* carried in the watermelon cultivar ‘ZXG01478’ (Zhang et al., 2013; Li et al., 2017). In another instance, Zhou and coworkers developed nine SSR markers tightly linked to the cucumber FW resistance gene *Foc-4*. Dong and group demonstrated five InDel markers for the main stabilization point of cucumber resistance *fw2.1* (Zhou et al., 2015; Dong et al., 2019); Wang and colleagues developed CAPS markers for two SNP sites of *Fom-2* and successfully converted them into functional markers (Wang, 2010). In this study, two InDel markers (3M9.425 and 3M16.689) were closely linked and separated with resistance genes *Fob1(t)*, which were clearly polymorphous. Further, the traditional agarose gel electrophoresis could effectively assist in the selection of positive individuals during breeding, overcoming the outstanding problems of low efficiency and poor accuracy of target character selection in traditional breeding for disease resistance. This study substantially provided scientific and technological support for breeding new varieties with disease resistance, thus promoting the breeding process of wax gourd for resistance to FW.

## Data Availability

The original contributions presented in this study are publicly available. The data sets presented in NCBI, PRJNA1264041.

## Supplementary material

Supplementary Figure 1 Marker 3M9.425 detection in 2.5% agarose gel;

Supplementary Figure 2 Marker 3M16.869 detection in 2.5% agarose gel;

Supplementary Table 1 Primer list.
