# Supplementary figures and images for: Fine mapping and characterization of Fusarium wilt (Fusarium oxysporum f. sp. benincasae) resistance gene Fob1(t) in wax gourd (Benincasa hispida Cogn.)
Source: Front Plant Sci. 2025 May 26;16:1555316. doi: 10.3389/fpls.2025.1555316 (PMC12146367; doi:10.3389/fpls.2025.1555316)

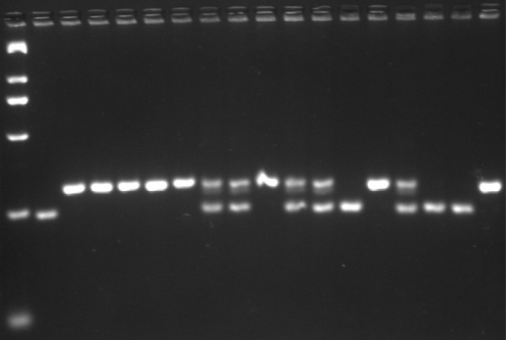

Supplement: Supplementary Figure 1 — Marker 3M9.425 detection in 2.5% agarose gel; [file Image1.tif]

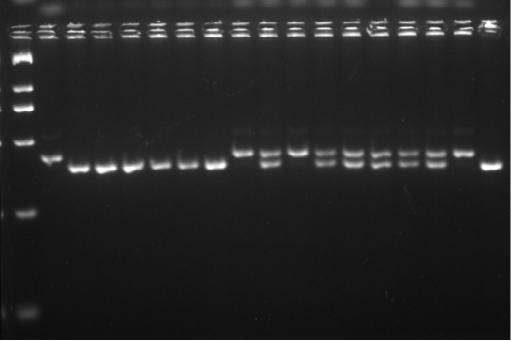

Supplement: Supplementary Figure 2 — Marker 3M16.869 detection in 2.5% agarose gel; [file Image2.tif]

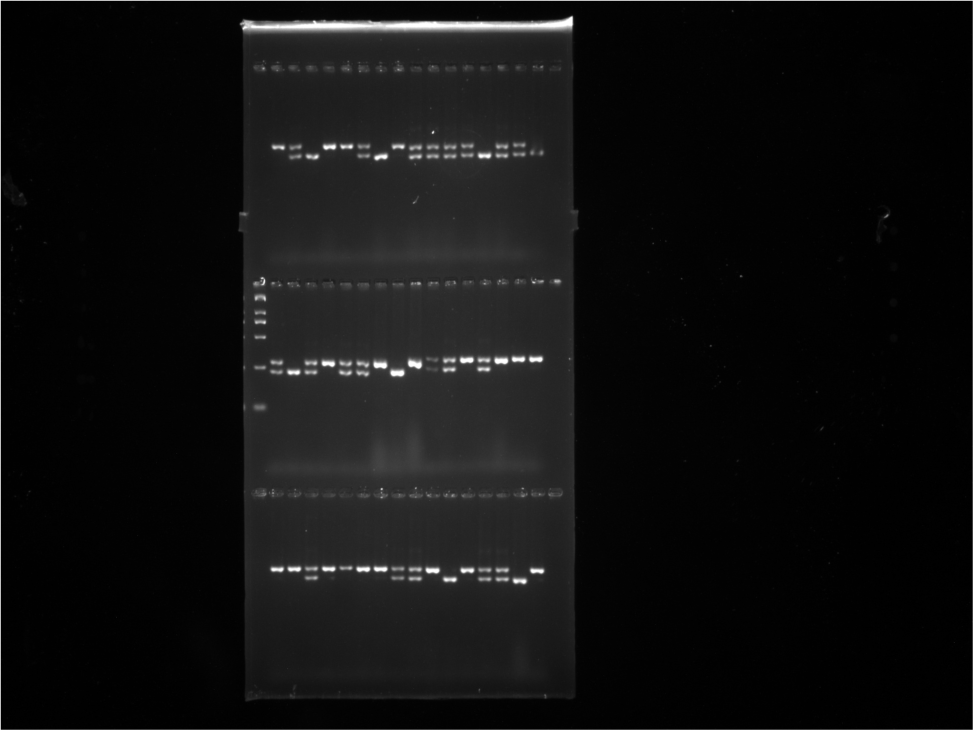

Supplement: Supplementary file 5 [file DataSheet1.zip › Original gel images-1/1M100.03-1.tif]

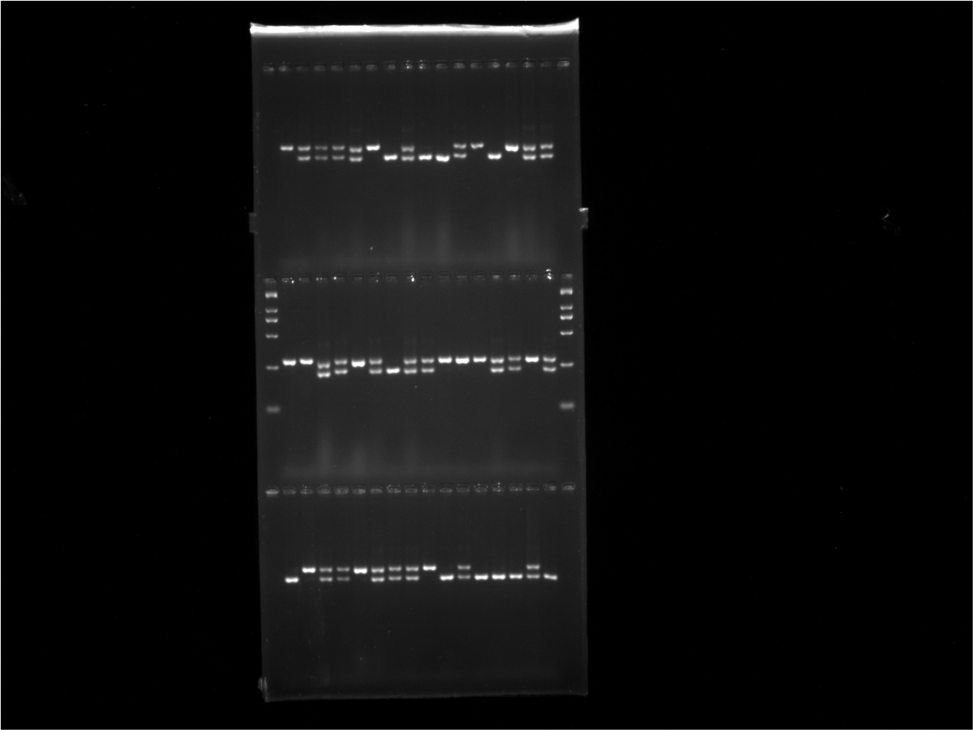

Supplement: Supplementary file 5 [file DataSheet1.zip › Original gel images-1/1M100.03-2.tif]

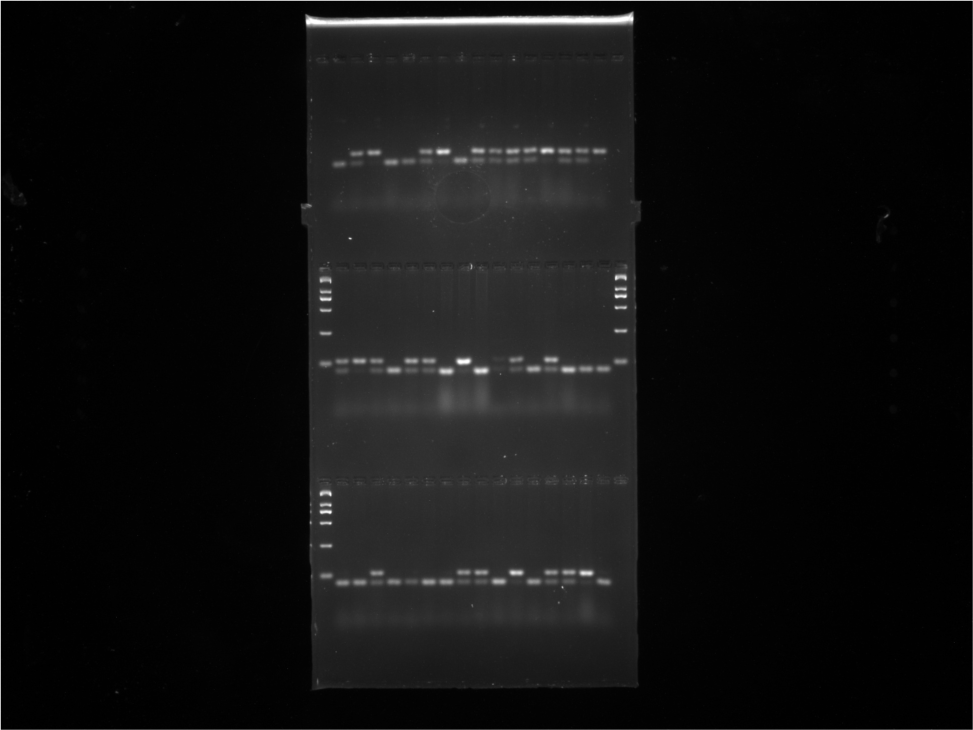

Supplement: Supplementary file 5 [file DataSheet1.zip › Original gel images-1/1M101.13-1.tif]

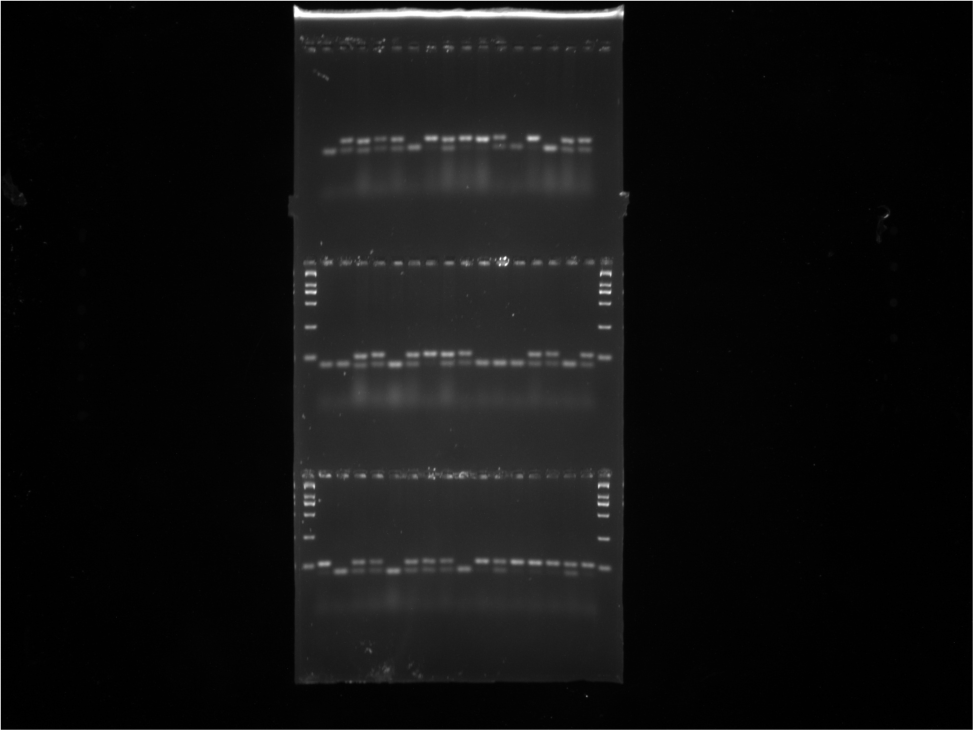

Supplement: Supplementary file 5 [file DataSheet1.zip › Original gel images-1/1M101.13-2.tif]

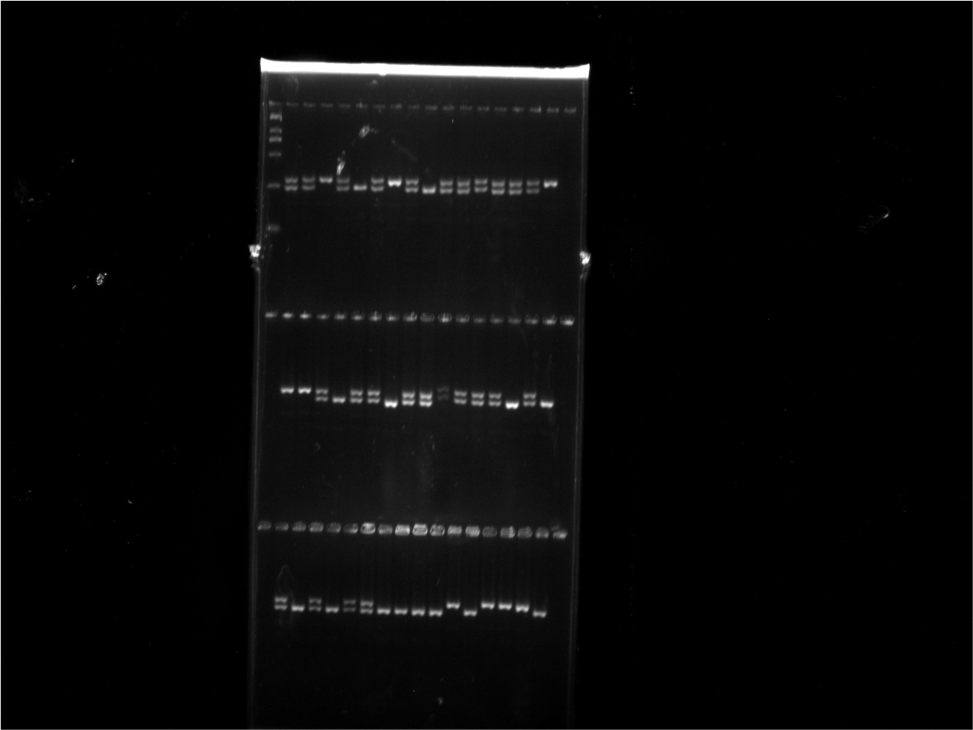

Supplement: Supplementary file 5 [file DataSheet1.zip › Original gel images-1/1M90.10-1.tif]

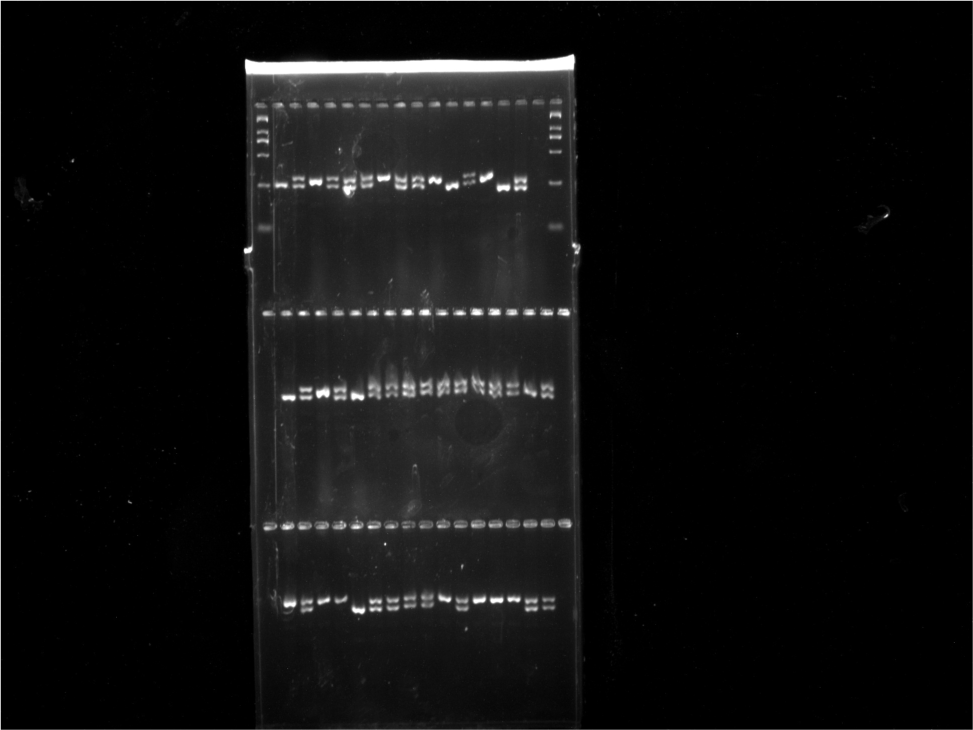

Supplement: Supplementary file 5 [file DataSheet1.zip › Original gel images-1/1M90.10-2.tif]

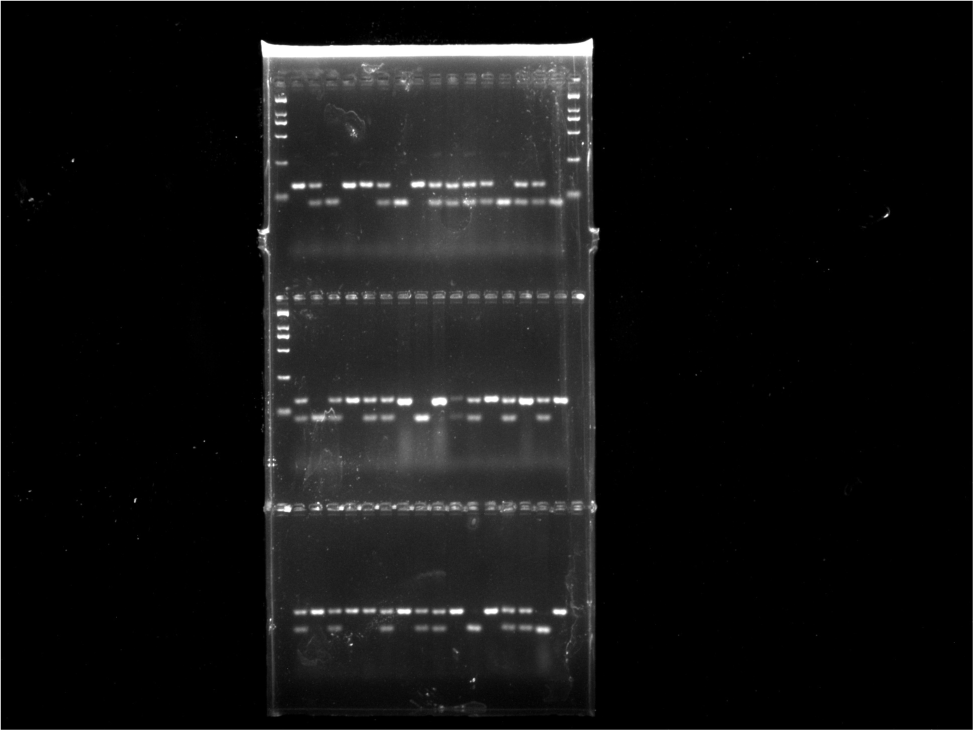

Supplement: Supplementary file 5 [file DataSheet1.zip › Original gel images-1/1M96.13-1.tif]

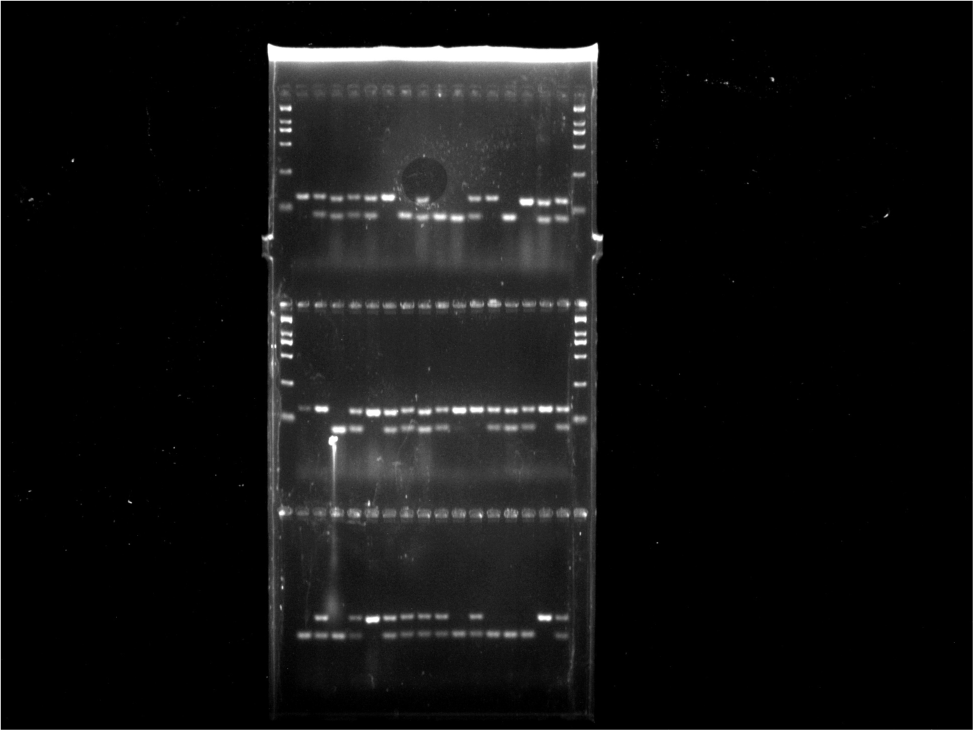

Supplement: Supplementary file 5 [file DataSheet1.zip › Original gel images-1/1M96.13-2.tif]

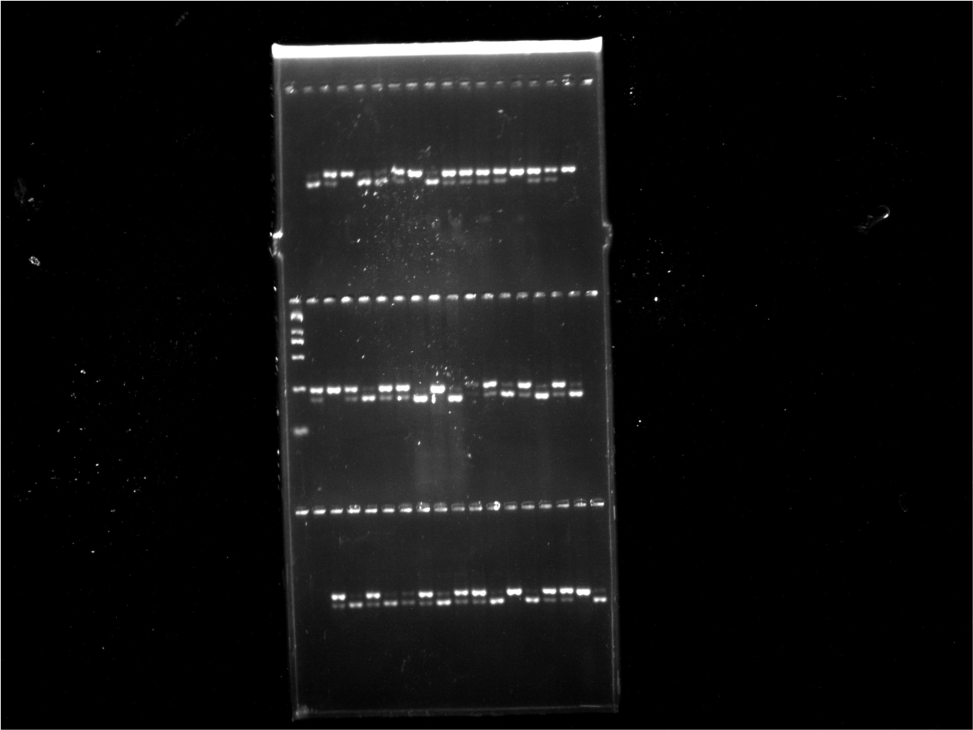

Supplement: Supplementary file 5 [file DataSheet1.zip › Original gel images-1/1M97.03-1.tif]

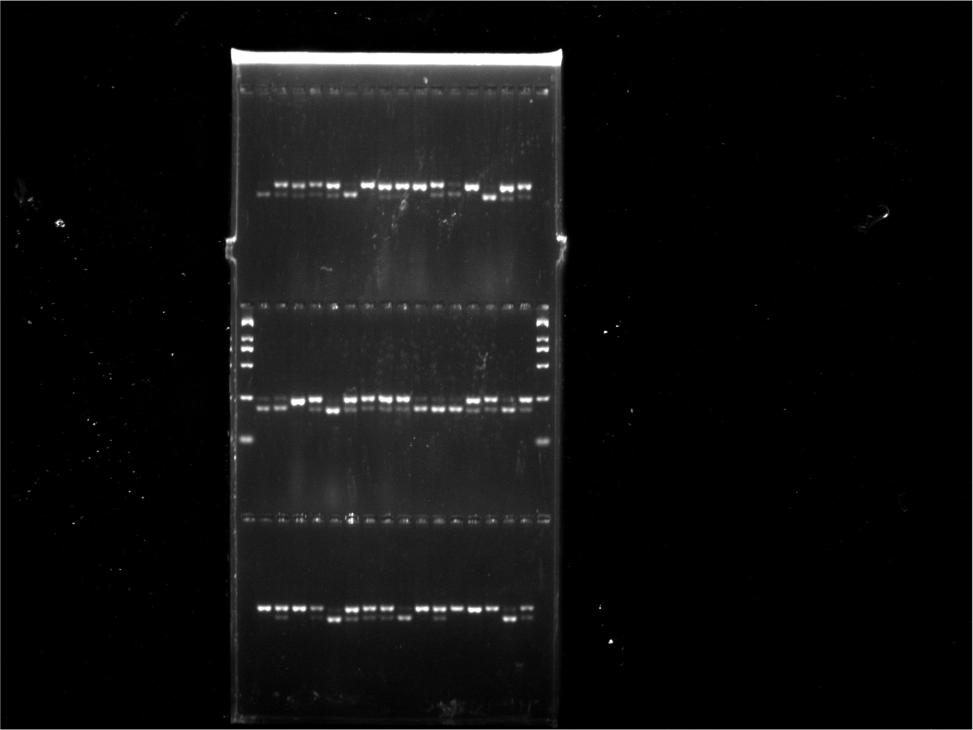

Supplement: Supplementary file 5 [file DataSheet1.zip › Original gel images-1/1M97.03-2.tif]

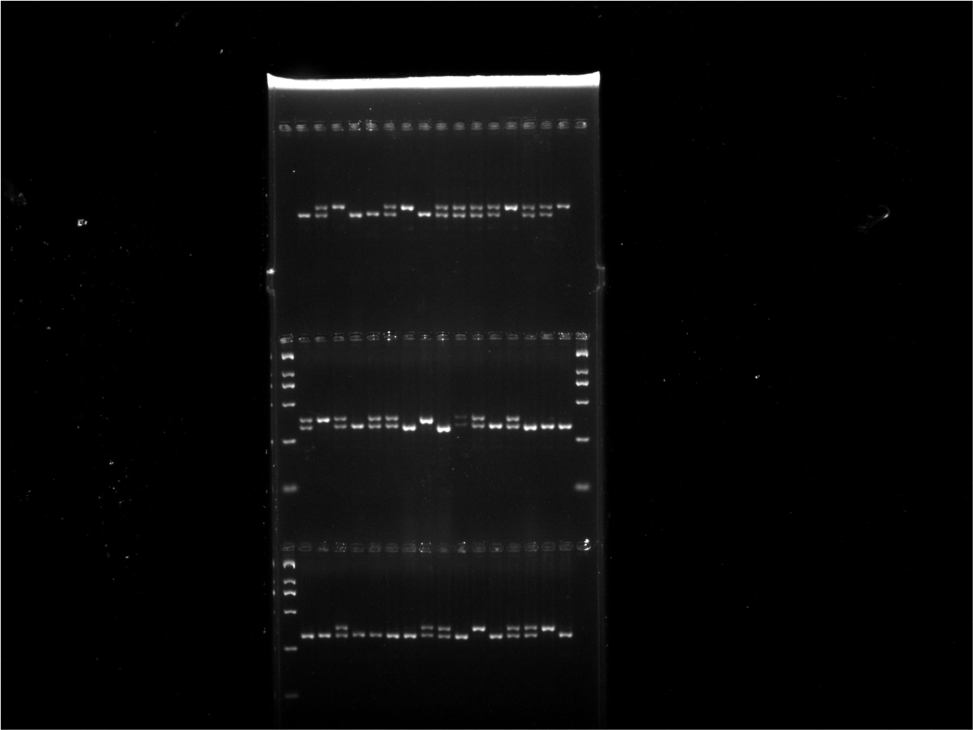

Supplement: Supplementary file 5 [file DataSheet1.zip › Original gel images-1/1M99.00-1.tif]

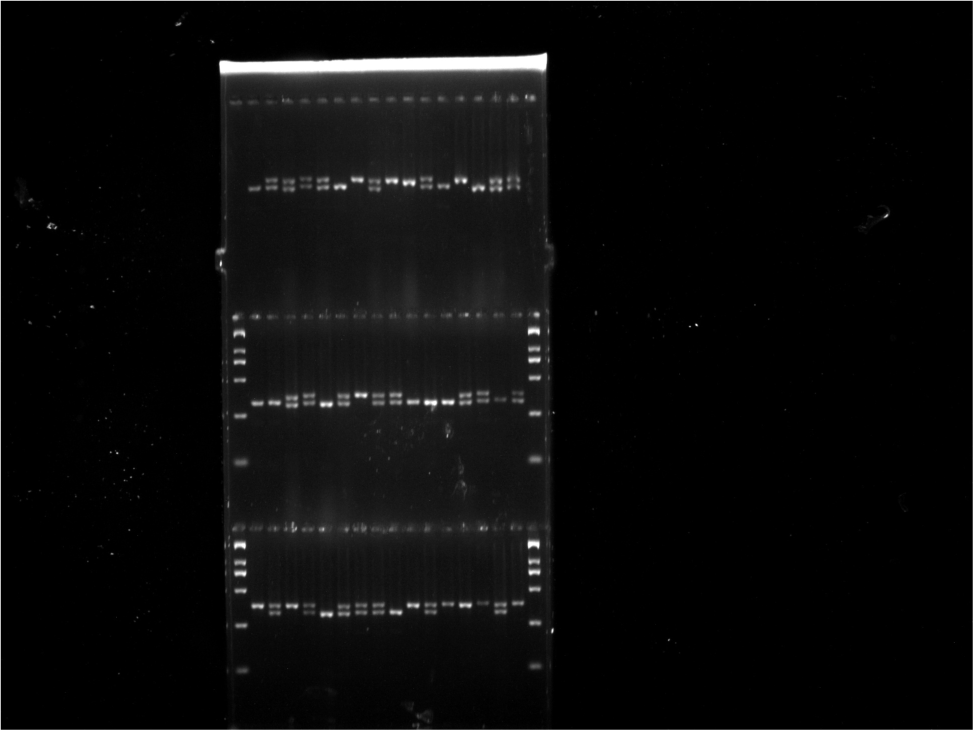

Supplement: Supplementary file 5 [file DataSheet1.zip › Original gel images-1/1M99.00-2.tif]

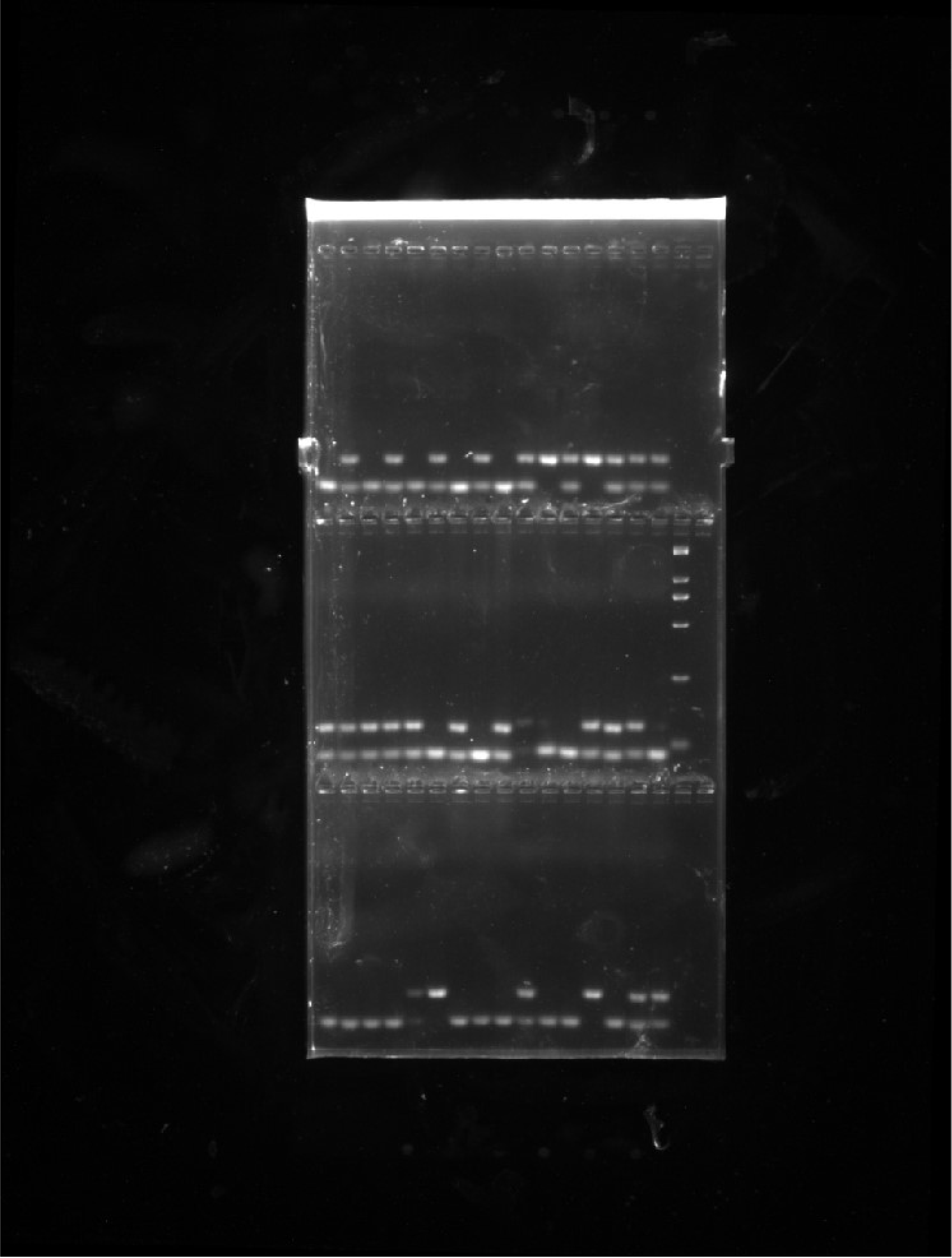

Supplement: Supplementary file 5 [file DataSheet1.zip › Original gel images-1/3M13.385-1.tif]

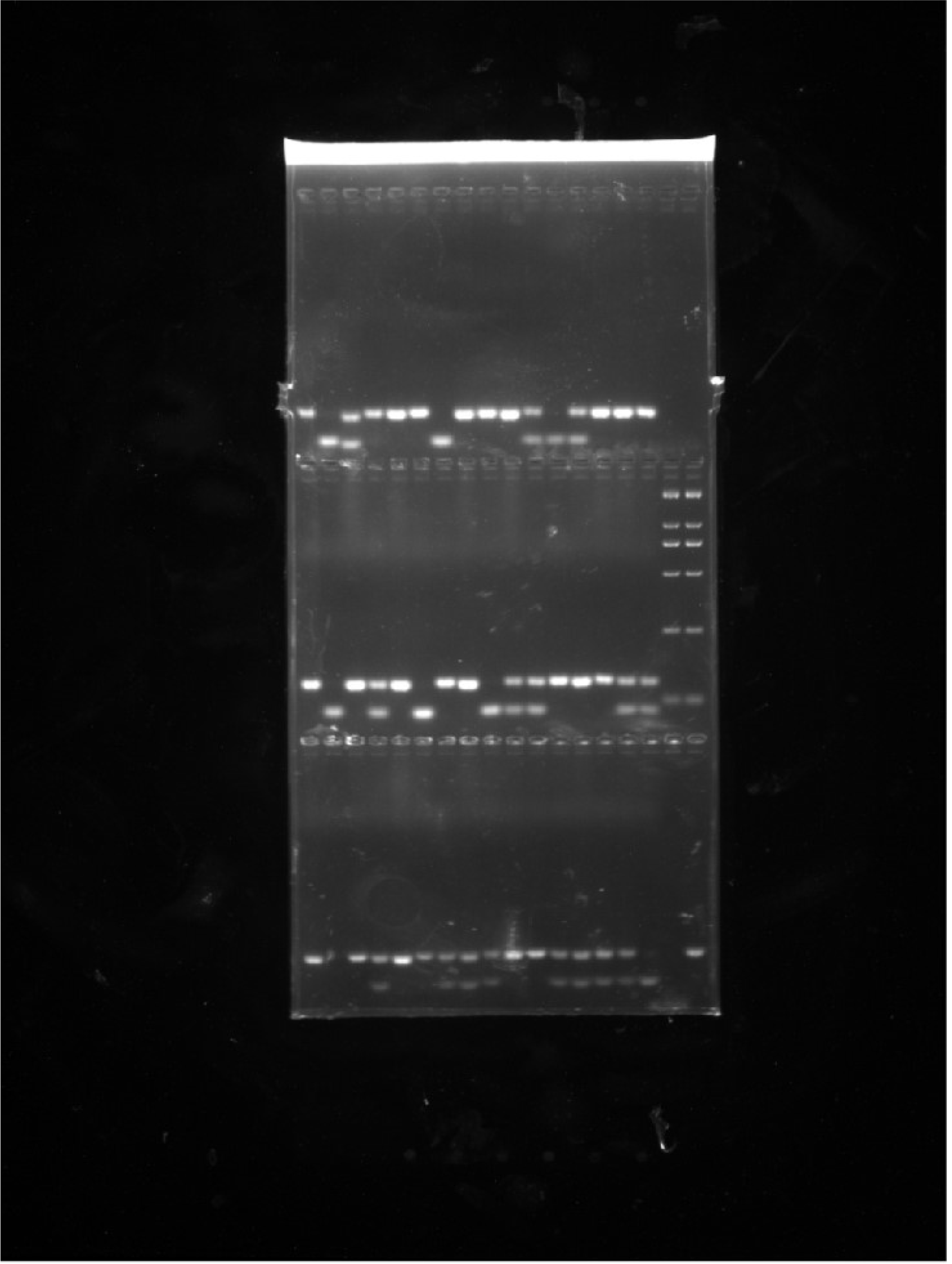

Supplement: Supplementary file 5 [file DataSheet1.zip › Original gel images-1/3M13.385-2.tif]

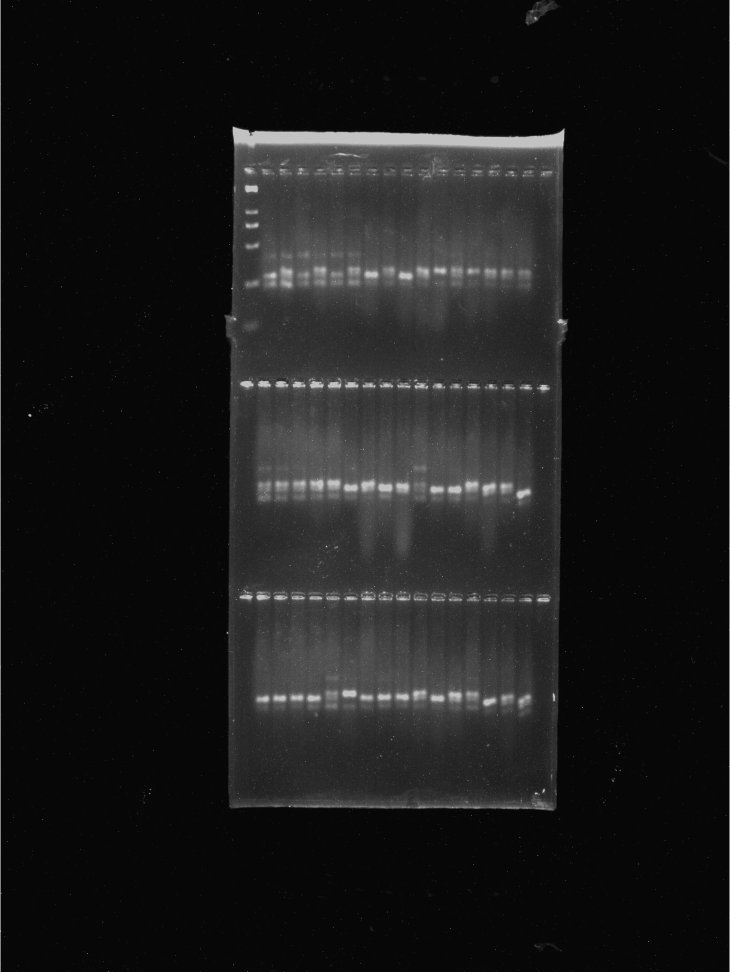

Supplement: Supplementary file 5 [file DataSheet1.zip › Original gel images-1/3M14.244-1.tif]

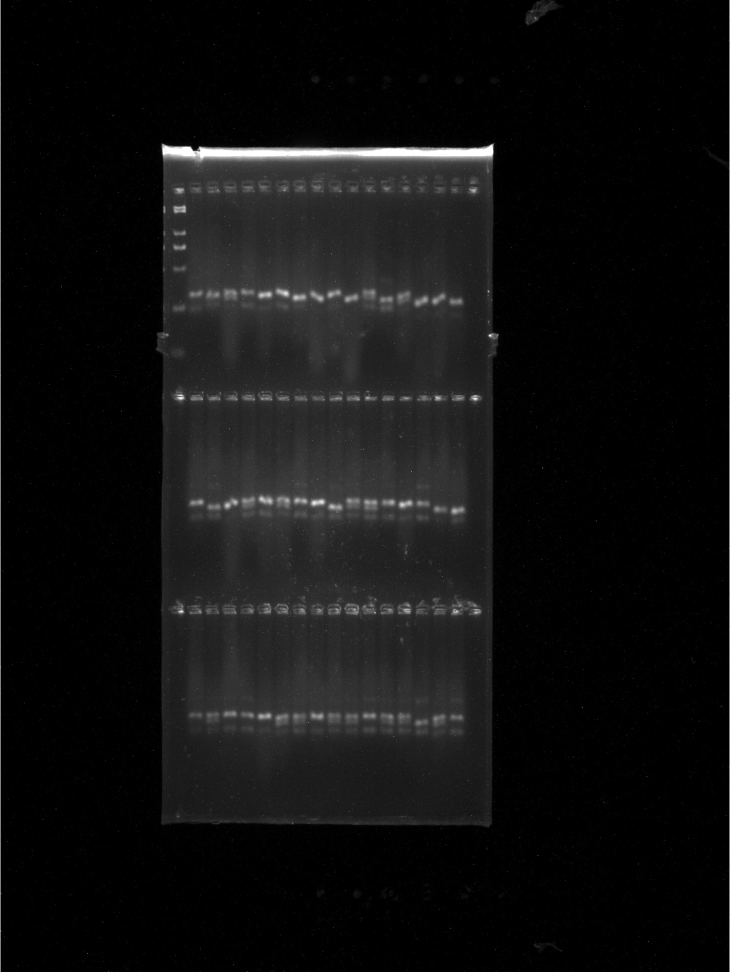

Supplement: Supplementary file 5 [file DataSheet1.zip › Original gel images-1/3M14.244-2.tif]

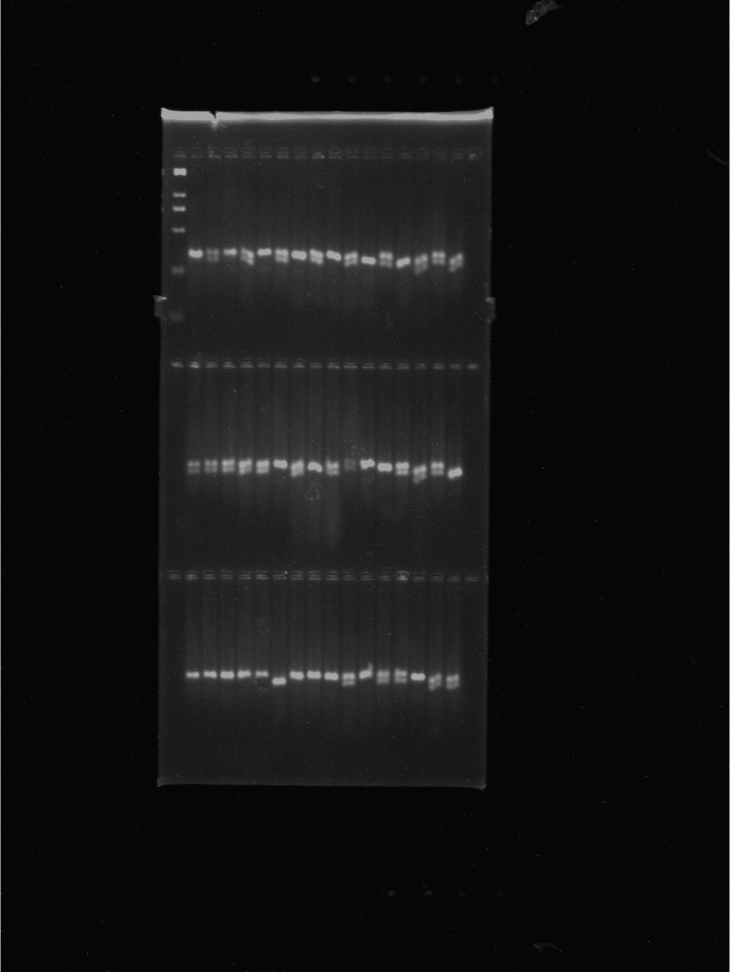

Supplement: Supplementary file 5 [file DataSheet1.zip › Original gel images-1/3M14.893-1.tif]

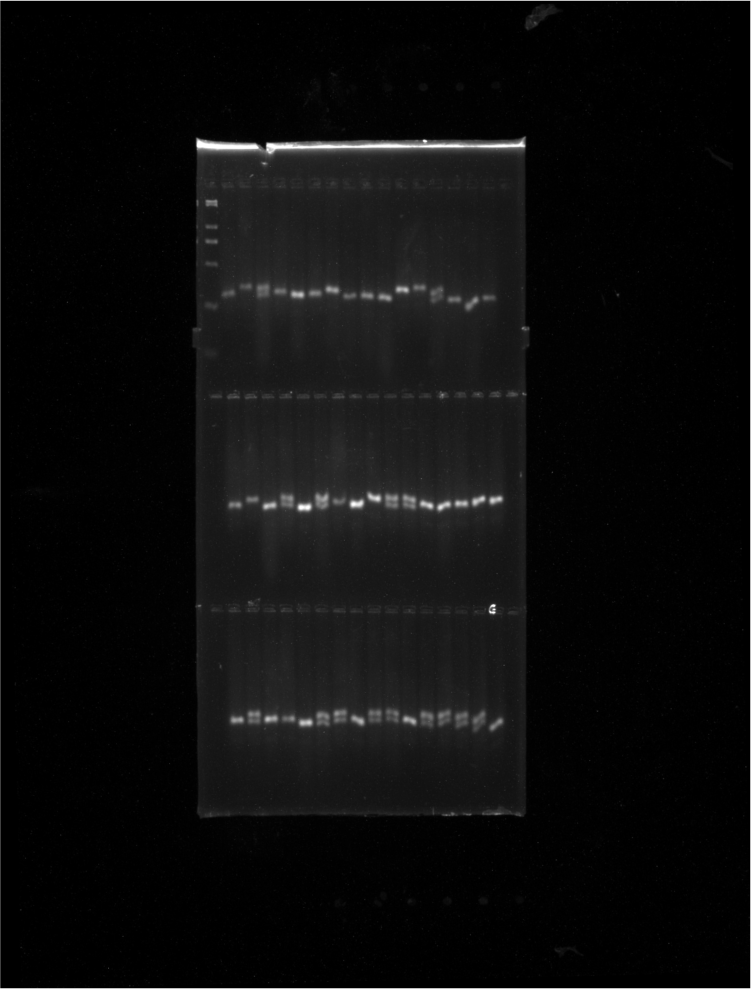

Supplement: Supplementary file 5 [file DataSheet1.zip › Original gel images-1/3M14.893-2.tif]

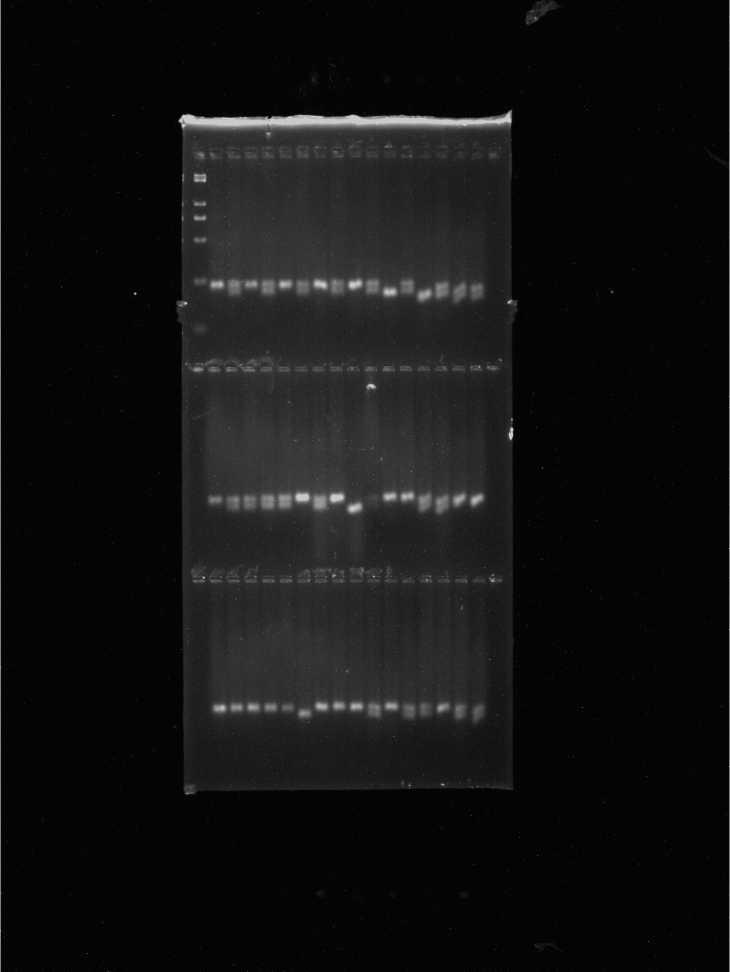

Supplement: Supplementary file 5 [file DataSheet1.zip › Original gel images-1/3M16.516-1.tif]

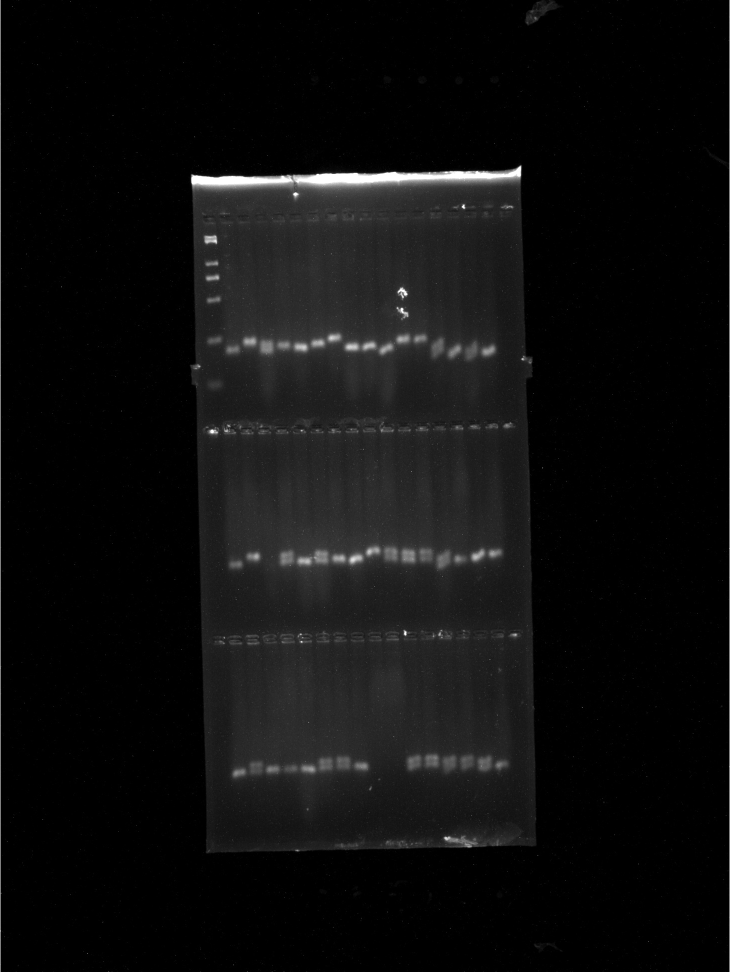

Supplement: Supplementary file 5 [file DataSheet1.zip › Original gel images-1/3M16.516-2.tif]

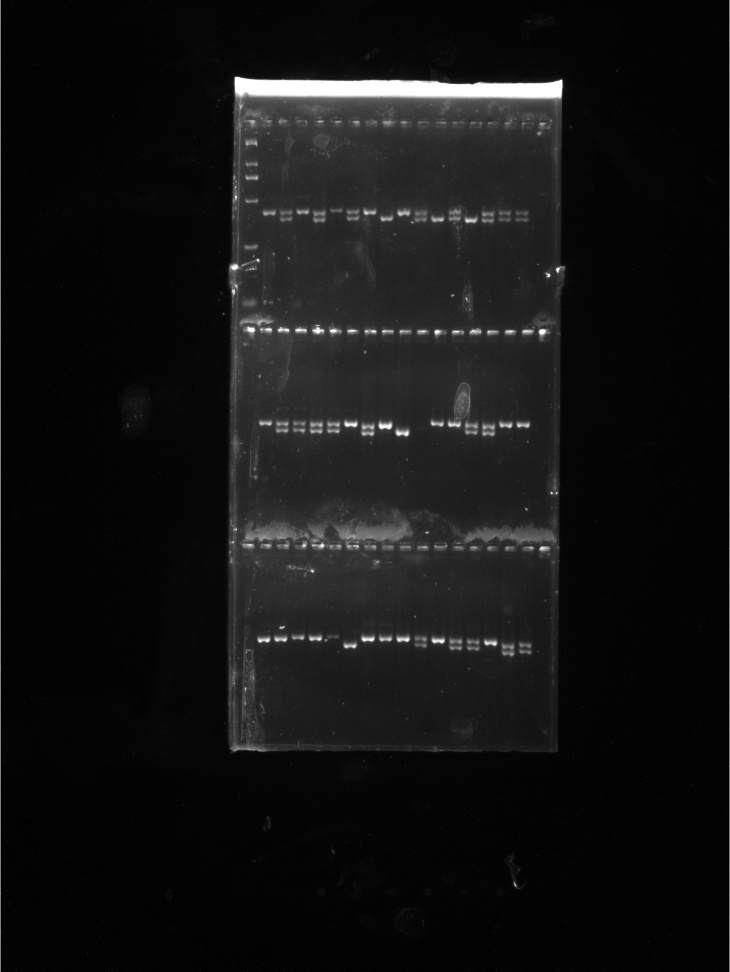

Supplement: Supplementary file 5 [file DataSheet1.zip › Original gel images-1/3M16.869-1.tif]

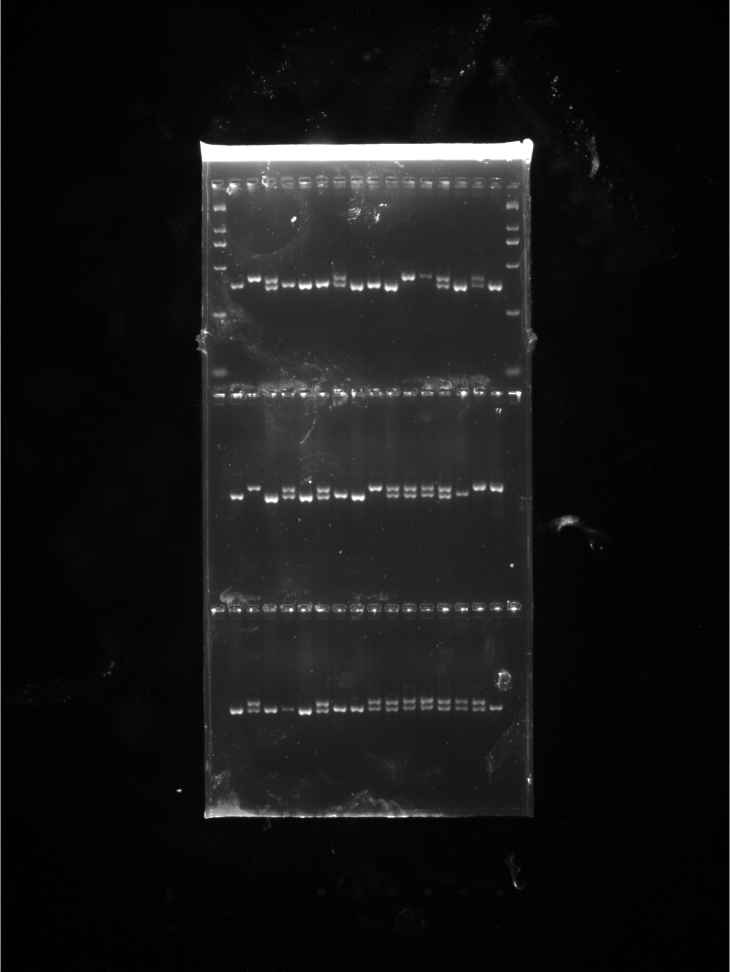

Supplement: Supplementary file 5 [file DataSheet1.zip › Original gel images-1/3M16.869-2.tif]

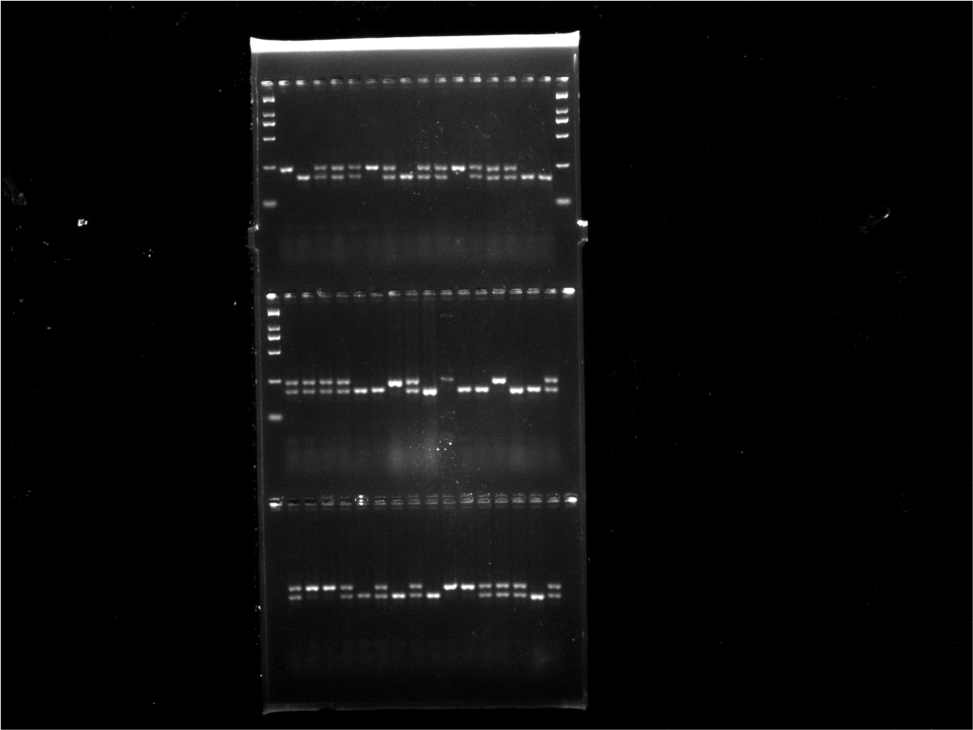

Supplement: Supplementary file 5 [file DataSheet1.zip › Original gel images-1/8M49.16 -1.tif]

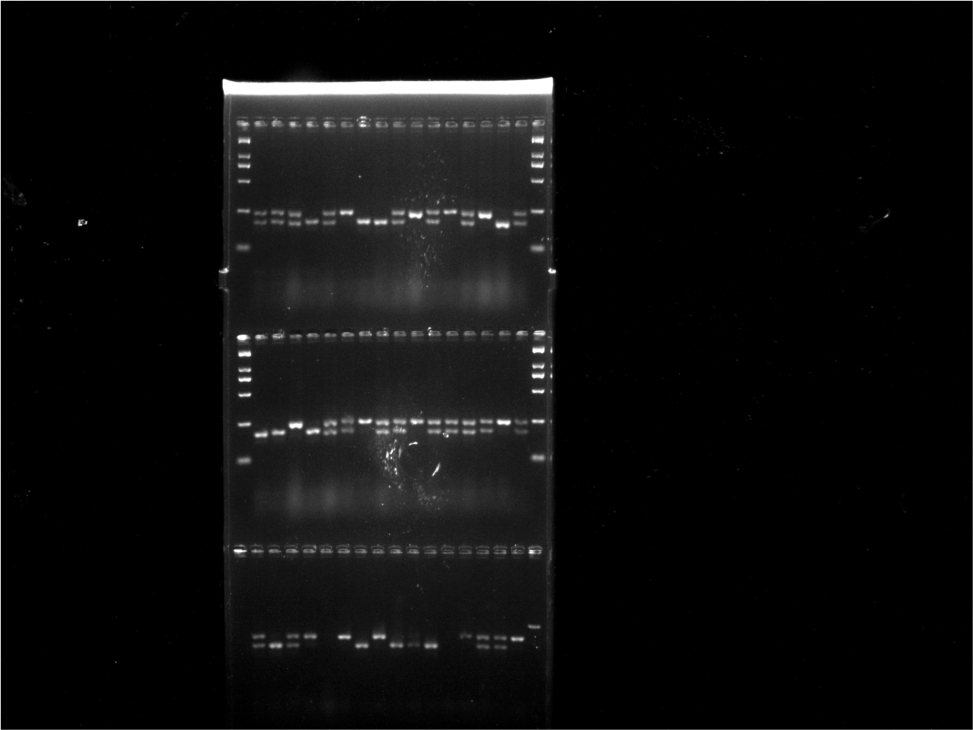

Supplement: Supplementary file 5 [file DataSheet1.zip › Original gel images-1/8M49.16 -2.tif]

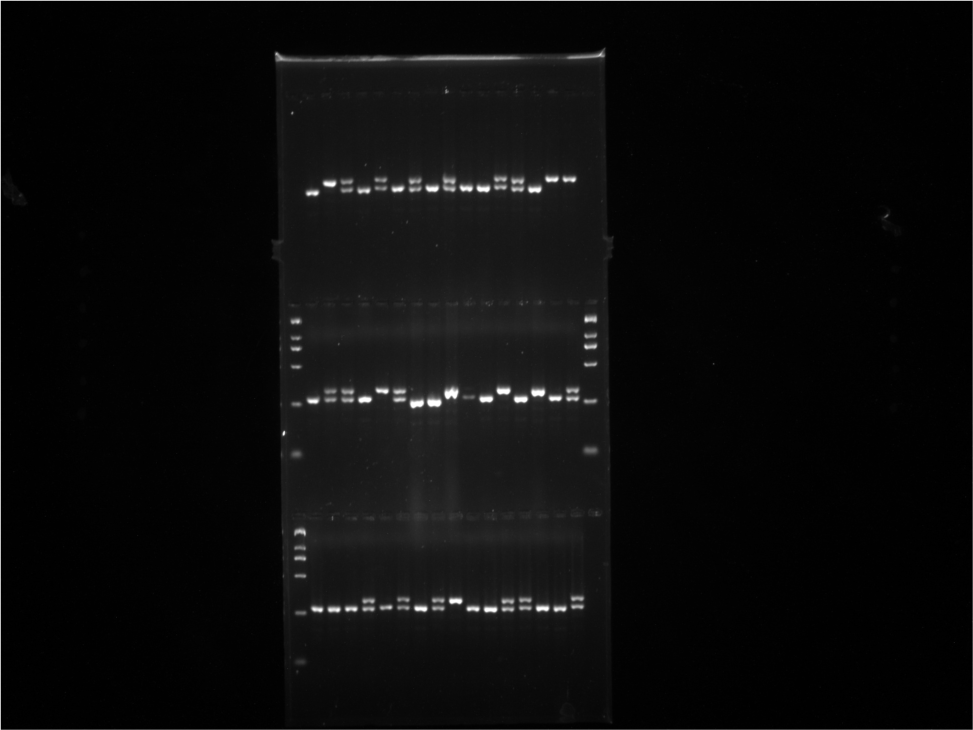

Supplement: Supplementary file 5 [file DataSheet1.zip › Original gel images-1/8M55.38-1.tif]

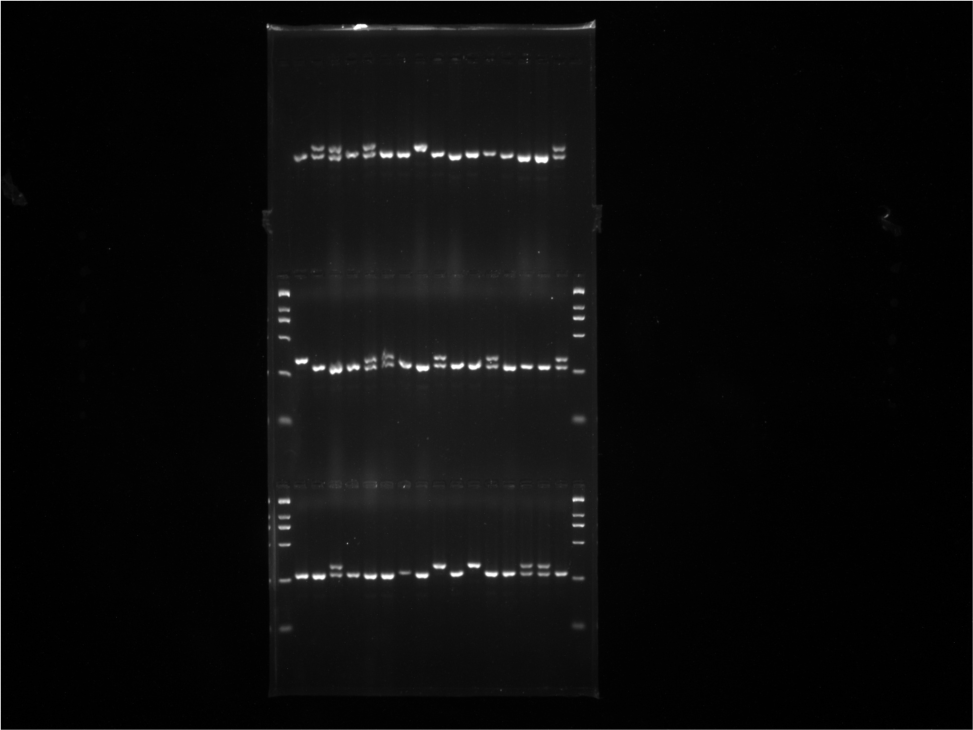

Supplement: Supplementary file 5 [file DataSheet1.zip › Original gel images-1/8M55.38-2.tif]

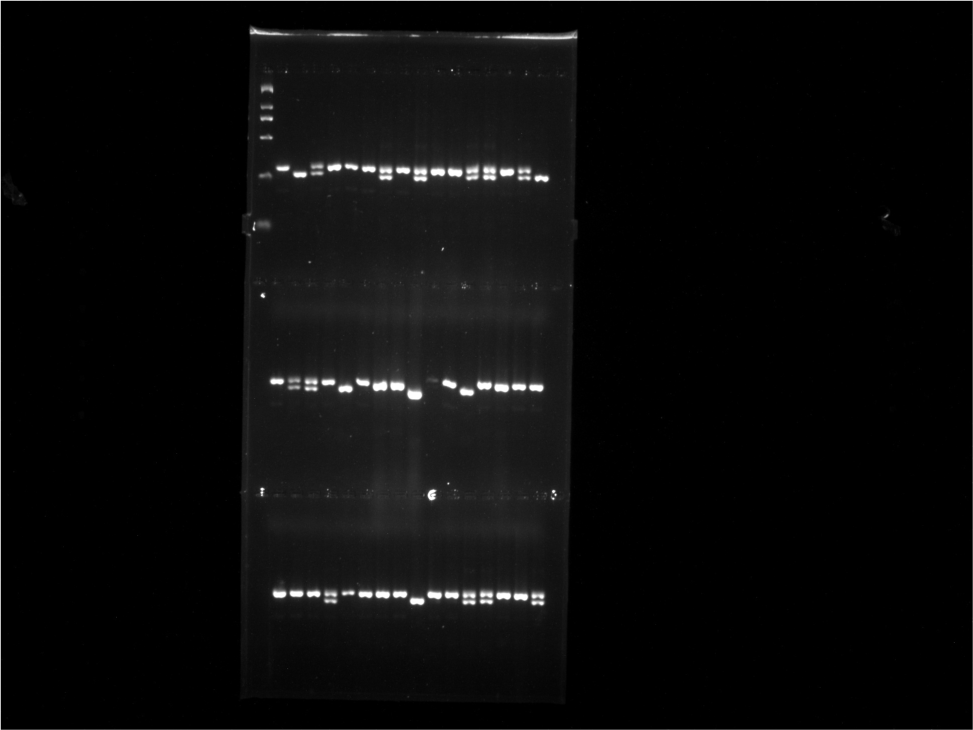

Supplement: Supplementary file 5 [file DataSheet1.zip › Original gel images-1/8M58.40-1.tif]

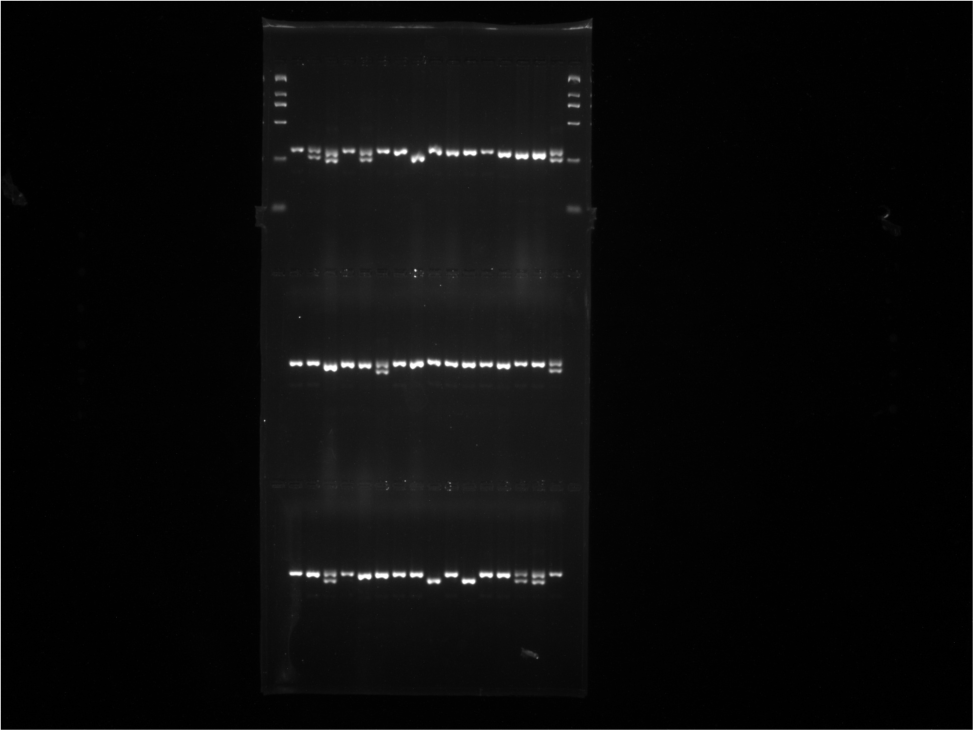

Supplement: Supplementary file 5 [file DataSheet1.zip › Original gel images-1/8M58.40-2.tif]

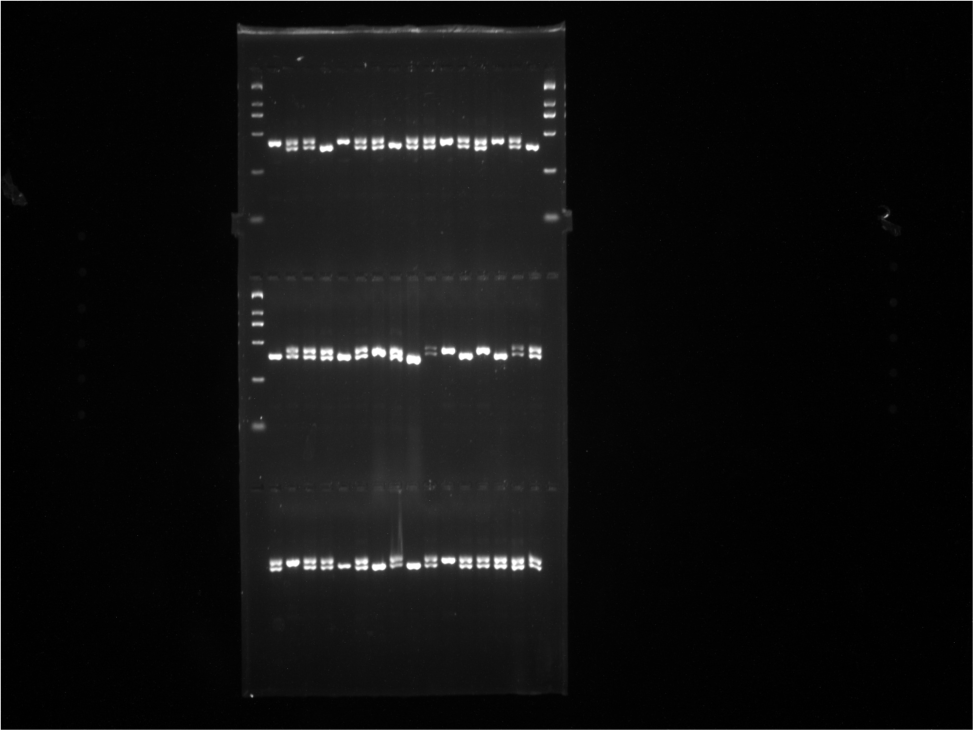

Supplement: Supplementary file 6 [file DataSheet2.zip › Original gel images-2/8M63.40-1.tif]

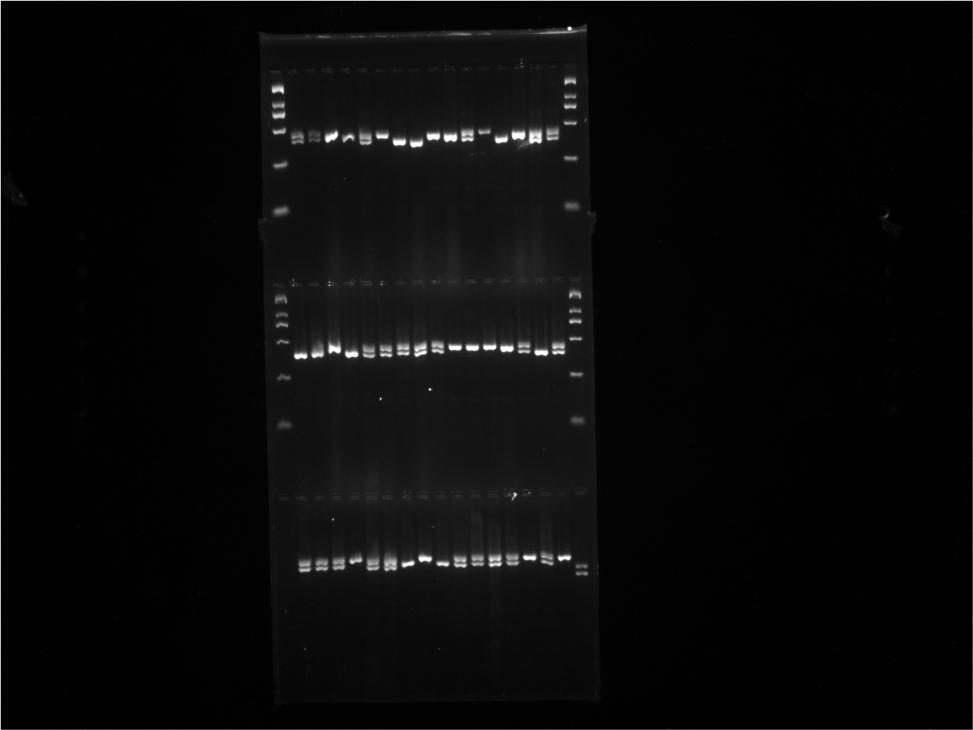

Supplement: Supplementary file 6 [file DataSheet2.zip › Original gel images-2/8M63.40-2.tif]

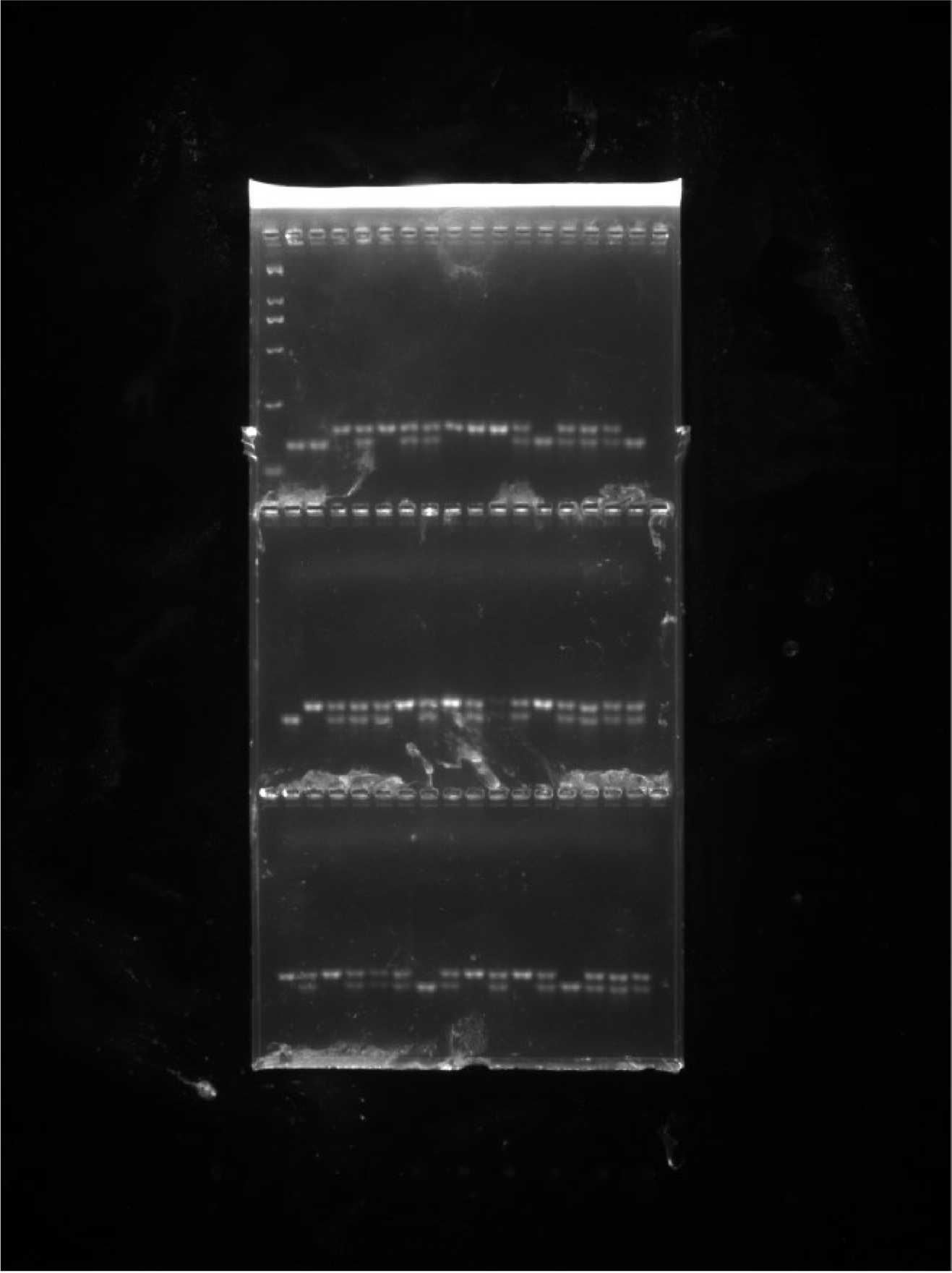

Supplement: Supplementary file 6 [file DataSheet2.zip › Original gel images-2/L1-1.tif]

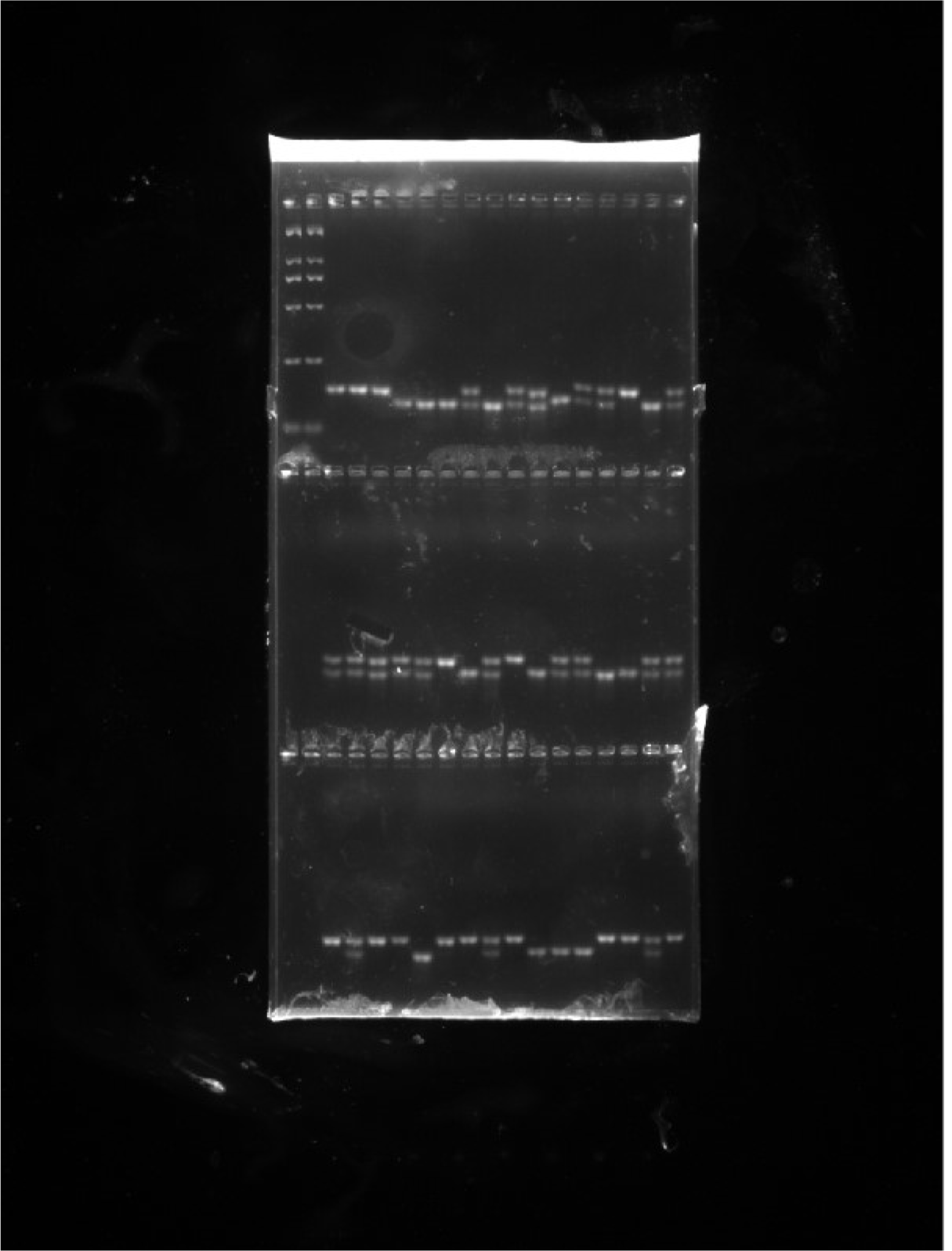

Supplement: Supplementary file 6 [file DataSheet2.zip › Original gel images-2/L1-2.tif]

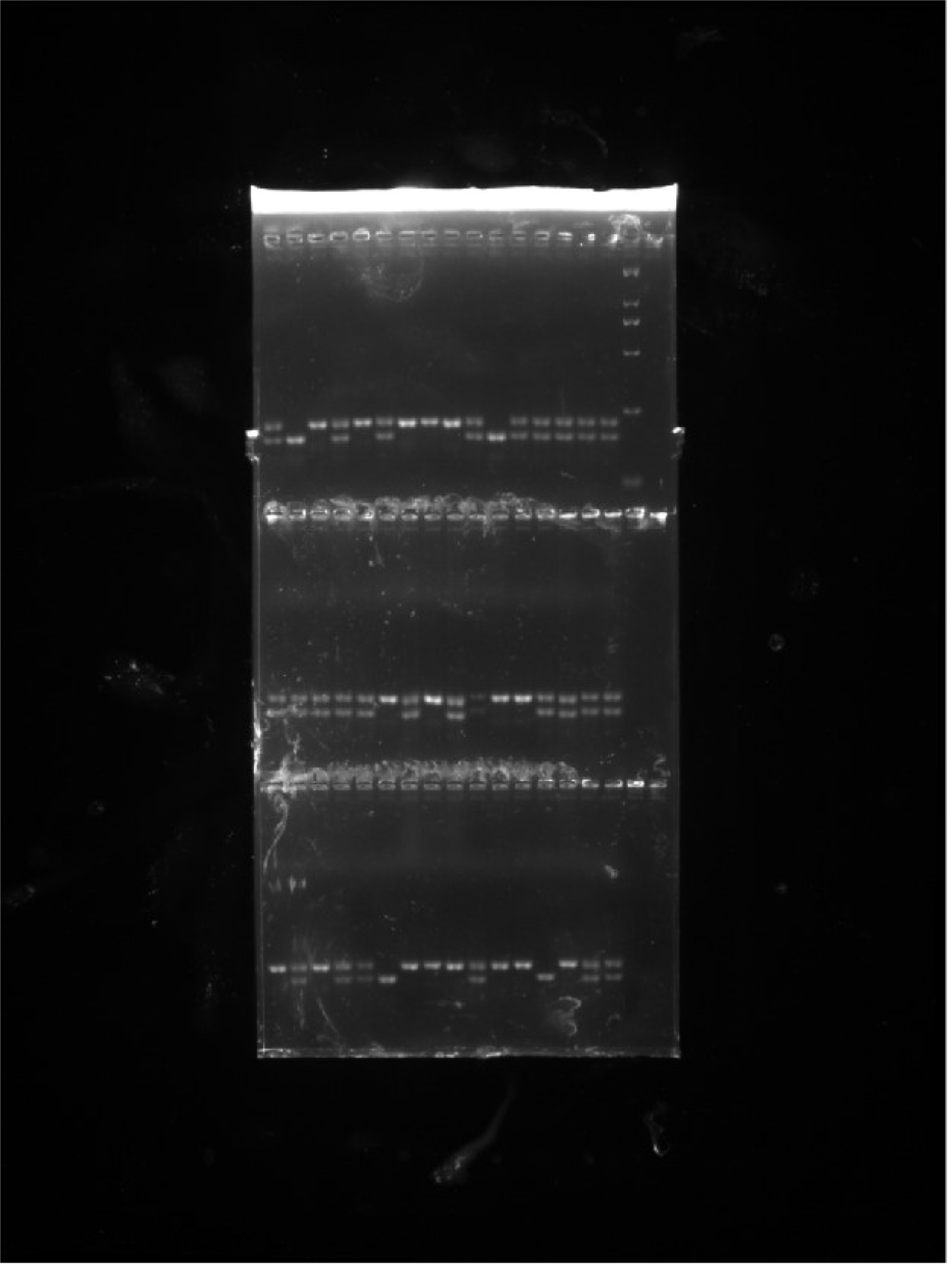

Supplement: Supplementary file 6 [file DataSheet2.zip › Original gel images-2/L2-1.tif]

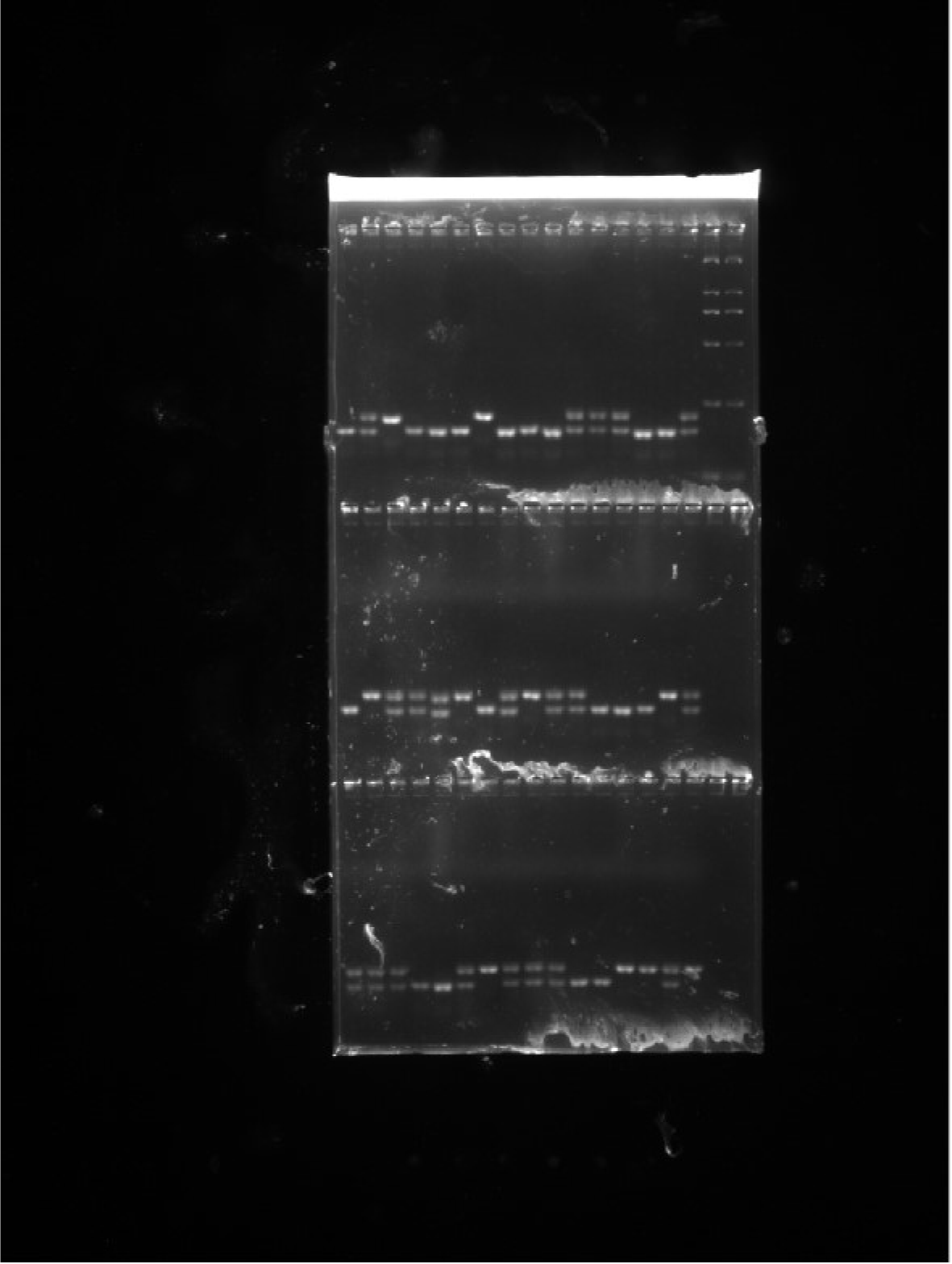

Supplement: Supplementary file 6 [file DataSheet2.zip › Original gel images-2/L2-2.tif]

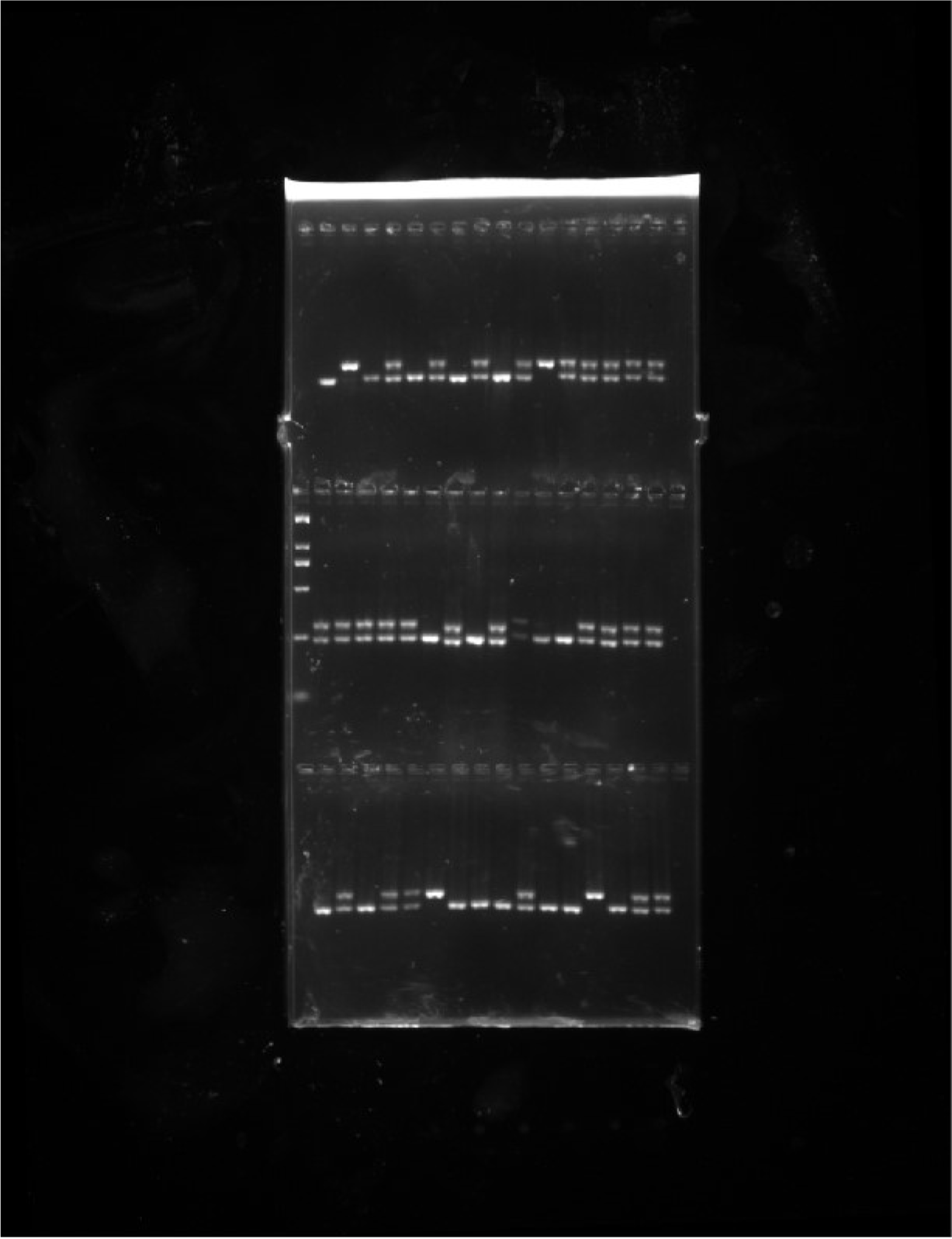

Supplement: Supplementary file 6 [file DataSheet2.zip › Original gel images-2/L3-1.tif]

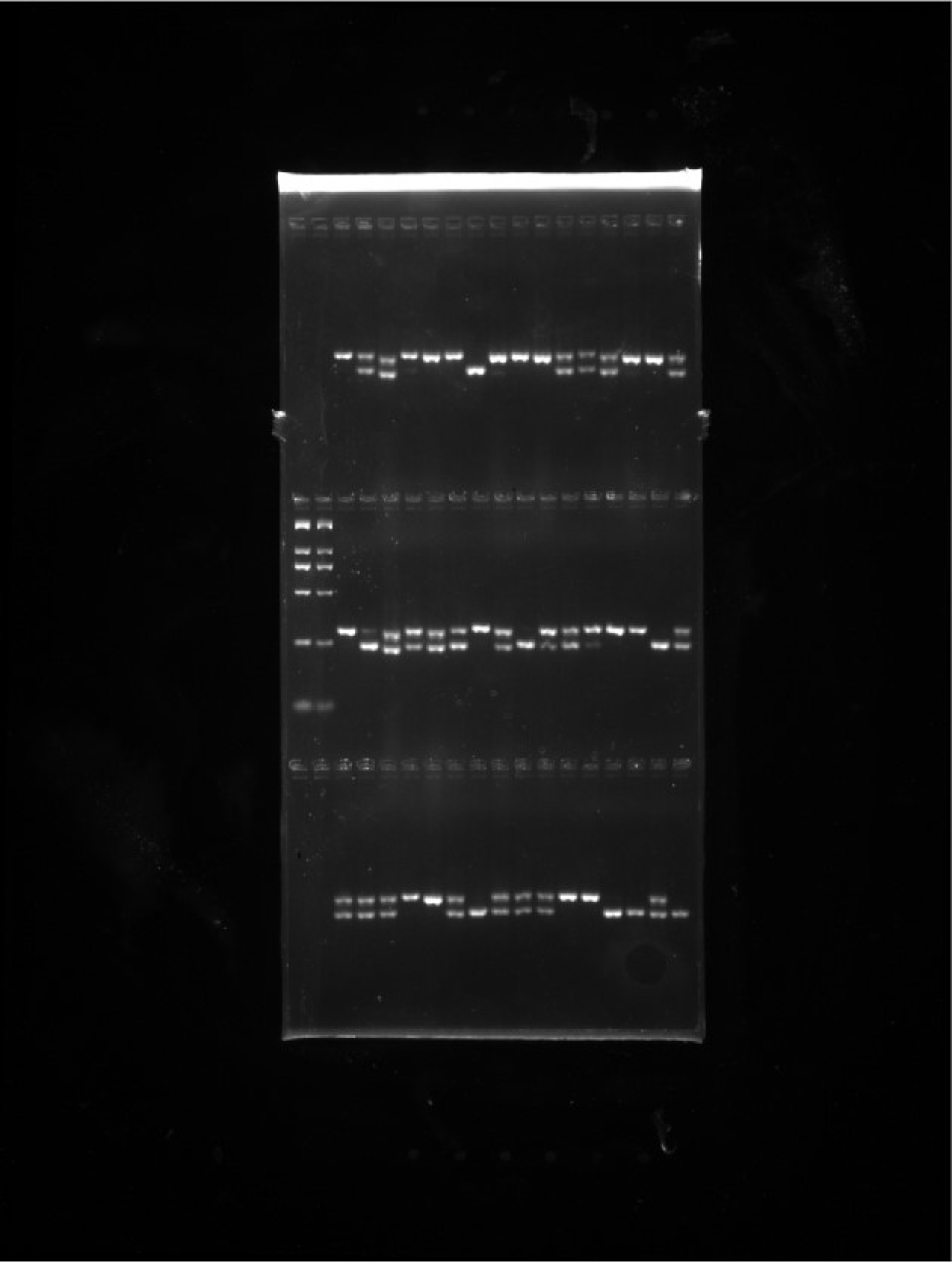

Supplement: Supplementary file 6 [file DataSheet2.zip › Original gel images-2/L3-2.tif]

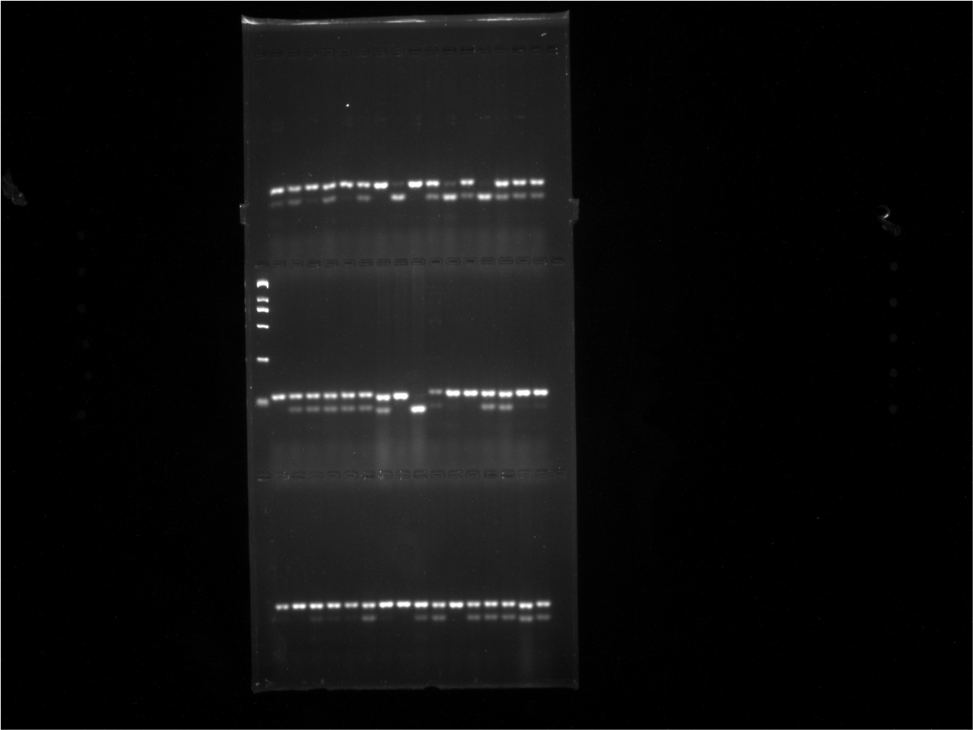

Supplement: Supplementary file 6 [file DataSheet2.zip › Original gel images-2/L4-1.tif]

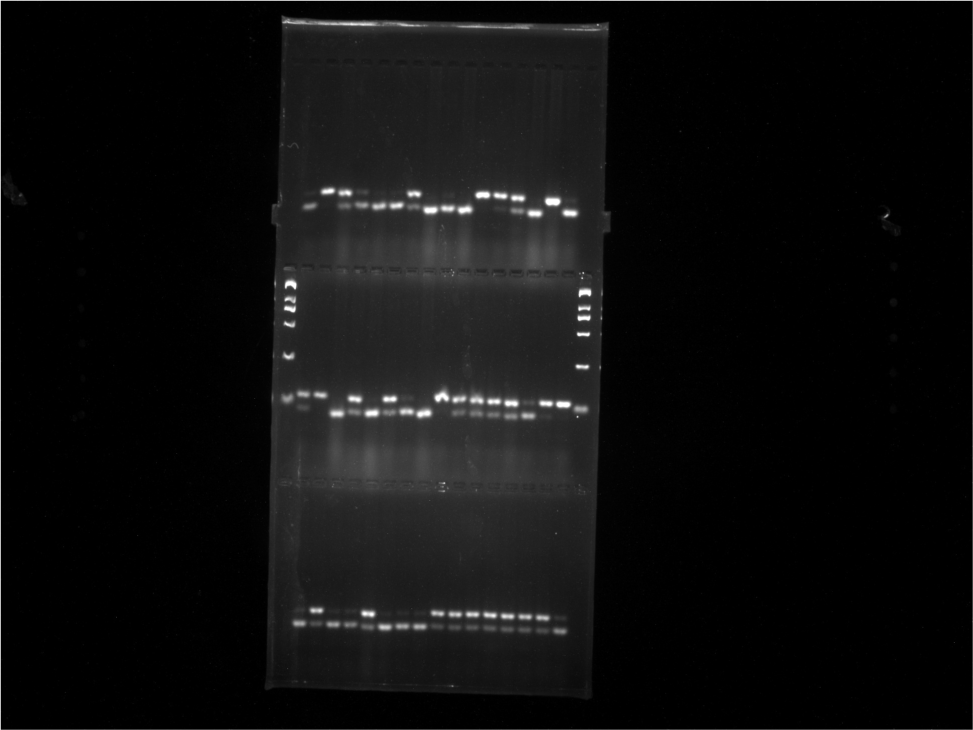

Supplement: Supplementary file 6 [file DataSheet2.zip › Original gel images-2/L4-2..tif]

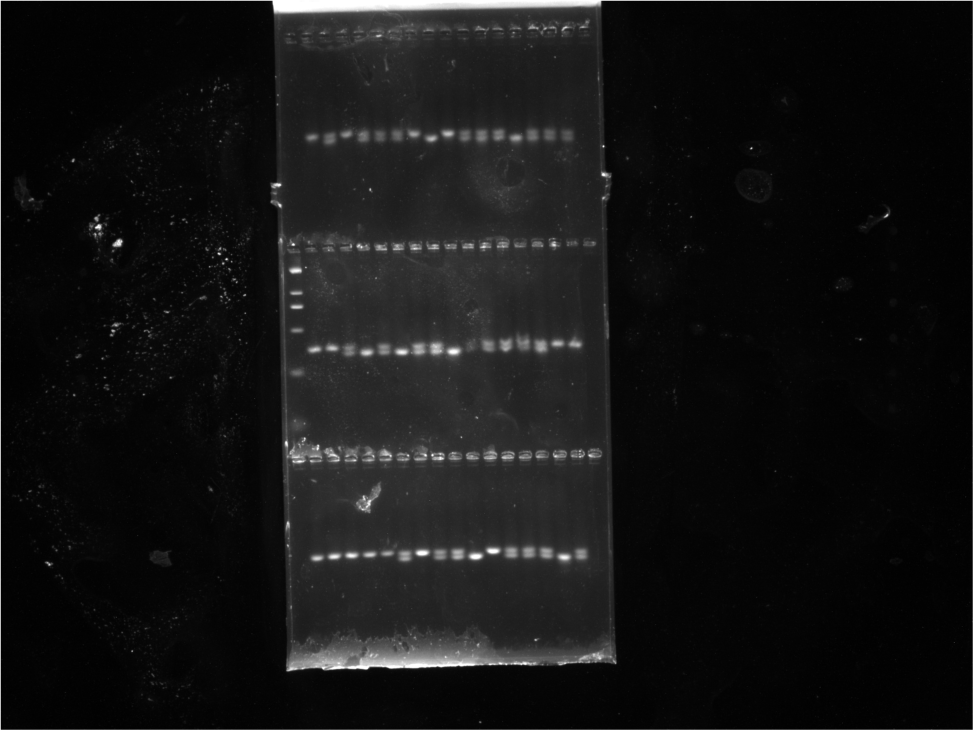

Supplement: Supplementary file 6 [file DataSheet2.zip › Original gel images-2/L5-1.tif]

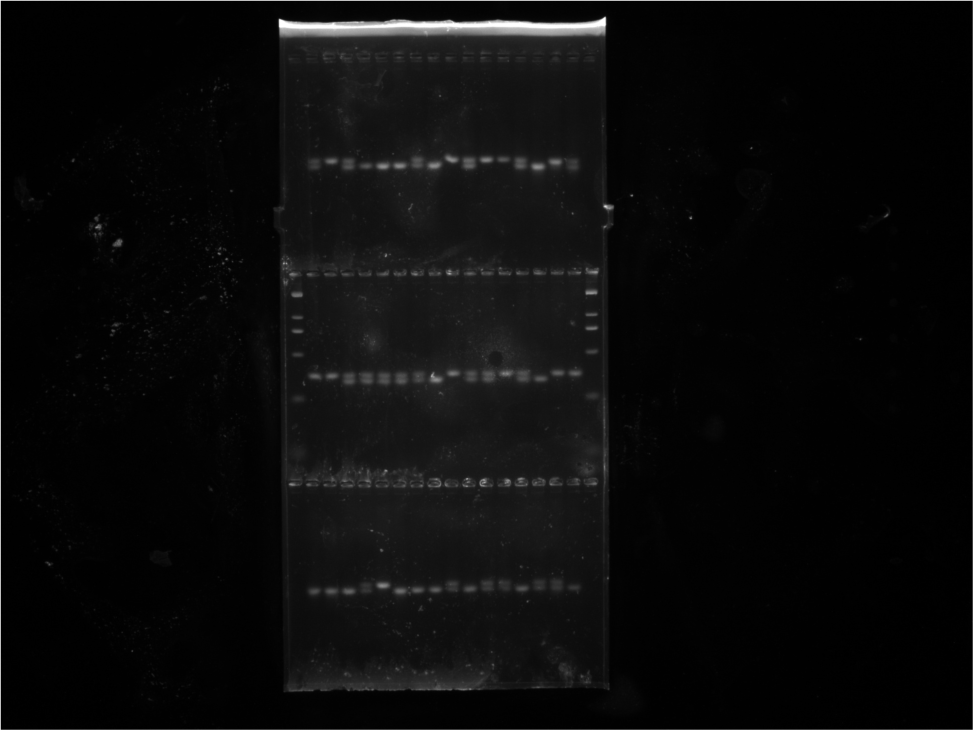

Supplement: Supplementary file 6 [file DataSheet2.zip › Original gel images-2/L5-2.tif]

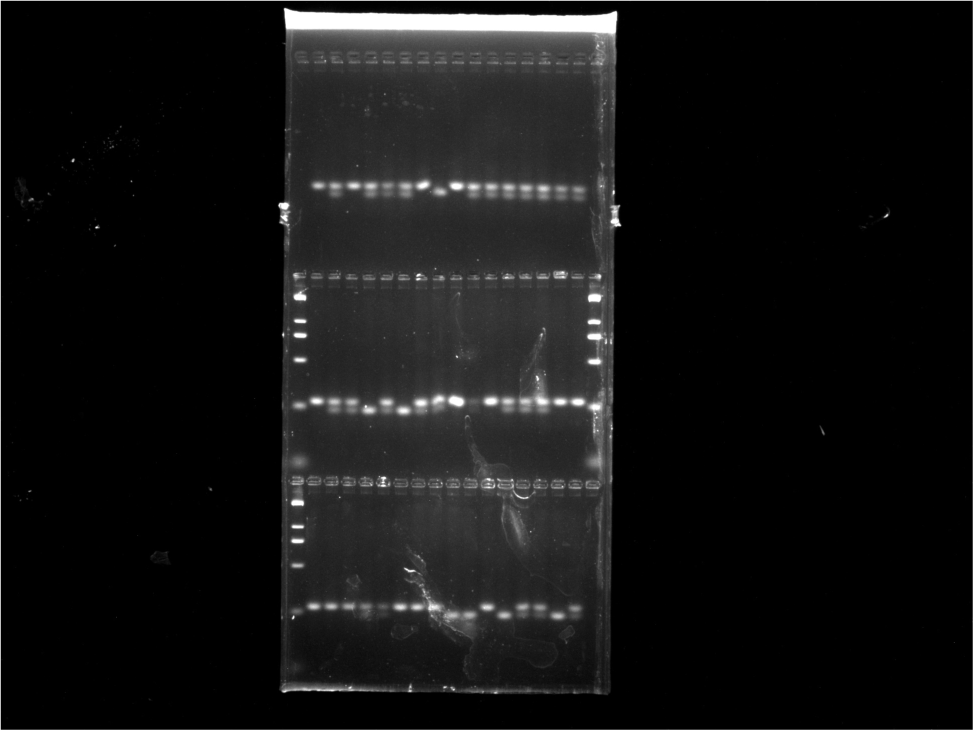

Supplement: Supplementary file 6 [file DataSheet2.zip › Original gel images-2/L6-1.tif]

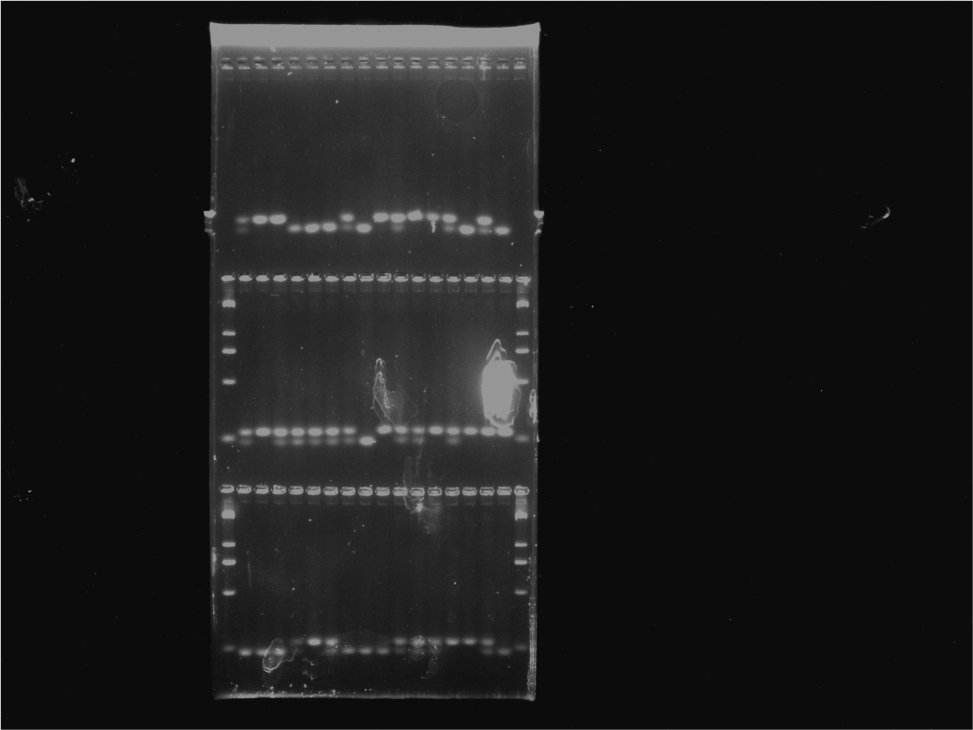

Supplement: Supplementary file 6 [file DataSheet2.zip › Original gel images-2/L6-2.tif]

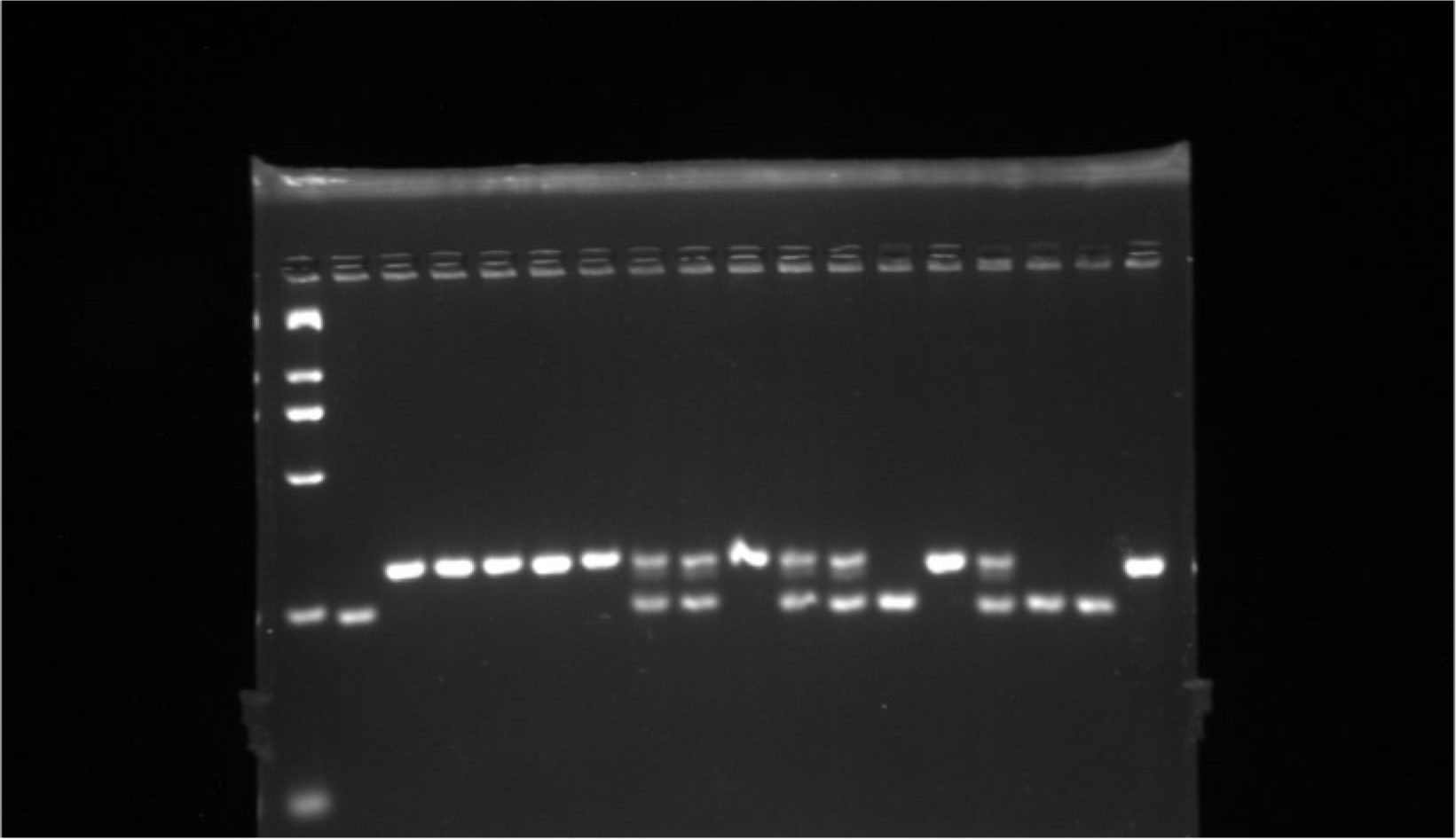

Supplement: Supplementary file 6 [file DataSheet2.zip › Original gel images-2/Supplementary Figure S1.tif]

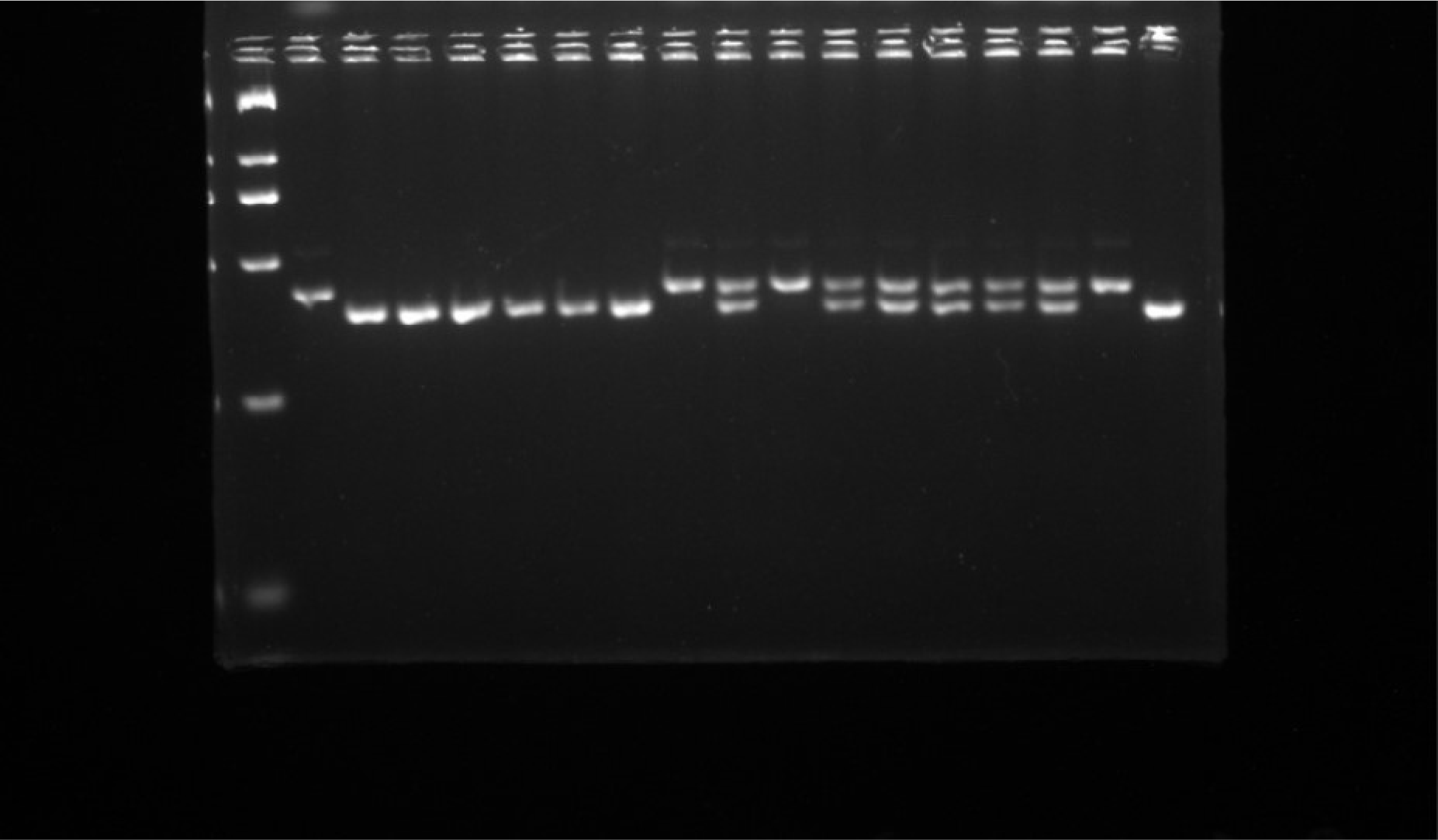

Supplement: Supplementary file 6 [file DataSheet2.zip › Original gel images-2/Supplementary Figure S2.tif]
